# Sensitivity analysis for the probability of benefit in randomized controlled trials with a binary treatment and a binary outcome

**DOI:** 10.1093/biostatistics/kxaf011

**Published:** 2025-06-02

**Authors:** Iuliana Ciocănea-Teodorescu, Erin E Gabriel, Arvid Sjölander

**Affiliations:** Victor Babeş National Institute of Pathology, Splaiul Independenţei 99-101, Bucharest 050096, Romania; Carol Davila University of Medicine and Pharmacy, Bulevardul Eroii Sanitari 8, Bucharest 050474, Romania; Section of Biostatistics, Department of Public Health, University of Copenhagen, Øster Farimagsgade 5, Copenhagen DK-1353, Denmark; Department of Medical Epidmiology and Biostatistics, Karolinska Institute, Stockholm 171 77, Sweden

**Keywords:** probability of benefit, randomized controlled trial, sensitivity analysis

## Abstract

For a comprehensive understanding of the effect of a given treatment on an outcome of interest, quantification of individual treatment heterogeneity is essential, alongside estimation of the average causal effect. However, even in randomized controlled trials, quantities such as the probability of benefit or the probability of harm are not identifiable, since multiple potential outcomes cannot be observed simultaneously for the same individual. We propose a sensitivity analysis for the probability of benefit in randomized controlled trial settings with a binary treatment and a binary outcome, by quantifying the deviation from conditional independence of the two potential outcomes, given a set of measured prognostic baseline covariates. We do this using a marginal sensitivity analysis parameter that does not depend on the number or complexity of the measured covariates. We provide a guide to estimation and interpretation, and illustrate our method in simulations, as well as using a real data example from a randomized controlled trial studying the effect of umbilical vein oxytocin administration on the need for manual removal of the placenta during birth.

## 1. INTRODUCTION

The gold standard for exploring causal mechanisms are randomized controlled trials. By randomizing the treatment, confounding bias can be eliminated, which allows investigators to focus on the causal relationship of interest. However, most randomized controlled trials are focused on estimating average causal effects, which are quantities that do not reflect individual treatment heterogeneity. In particular, a treatment may have no causal effect on an outcome on average, but may still benefit and harm a substantial proportion of the population, if the beneficial and harmful effects cancel out, on average. Identifying a relevantly high probability of benefit in this scenario constitutes motivation to perform further subgroup analyses, in an effort to characterize subpopulations which stand to profit from being treated (VanderWeele and Knol 2011).

Suppose the treatment is binary, and consider the two potential outcomes under treatment and control, respectively. The probability of benefit is the probability that the potential outcome of an individual, had they been treated, is more favorable than the potential outcome of that same individual, had they not been treated. Similarly, the probability of harm is the probability that the potential outcome of an individual, had they not been treated, is more favorable than the potential outcome of that same individual, had they been treated. Because we never observe both potential outcomes for an individual simultaneously, the probability of benefit and the probability of harm, which involve distributions of joint potential outcomes, are generally unidentifiable, even under randomization (Fay et al. 2018; Greenland et al. 2020). Therefore, if one is interested in these quantities, possible ways of proceeding include obtaining bounds or performing sensitivity analysis.

Bounds for the probability of benefit or the probability of harm in randomized controlled trial settings, have been developed in Fan and Park (2010), Huang et al. (2017), Tian and Pearl (2000), Gadbury et al. (2004), Gadbury and Iyer (2000) for binary, ordinal and continuous outcomes. Often, these bounds profit from the availability and incorporation of information on other baseline prognostic variables measured during the trial, or from additional assumptions placed on the joint distribution of the potential outcomes. Sensitivity analysis allows for these assumptions to be quantified via sensitivity parameters, which can be varied to gauge their impact on resulting target parameter estimates. Literature devoted to sensitivity analysis for the probability of benefit or related quantities, however, is limited. This includes Gadbury et al. (2001), who proposed to evaluate individual treatment heterogeneity by varying a correlation coefficient modeling the dependence of the two potential outcomes, and Zhang et al. (2013), who put forward two approaches. The first is a latent variable approach, and assumes the existence of an unmeasured variable $ U $, such that the two potential outcomes are independent conditional on $ U $ and any other measured baseline covariates. The sensitivity analysis is then based on varying assumptions concerning the distribution of $ U $. The second approach is based on specifying the dependence between the two potential outcomes within each level of the measured covariates directly, using the conditional odds ratio. However, the dimensionality of the sensitivity analysis parameter space, and hence the feasibility of the latter method, depends on the complexity of the measured covariates.

Our sensitivity analysis strategy is similar to that of Zhang et al. (2013), in that we aim to quantify the deviation from conditional independence between the potential outcomes, given available baseline covariates. However, we propose a sensitivity analysis parameter that is marginal, so that, regardless of the dimension of the baseline covariate space, only a single parameter is needed to identify the probability of benefit. We make no assumptions about the baseline covariates and their distribution, or about the nature of their association with the potential outcomes under treatment and control. In this paper, we focus on the probability of benefit as a target parameter, but our approach can be used for the probability of harm, or related quantities, such as the so-called *COST-parameters* introduced in Huitfeldt et al. (2018).

This paper is organized as follows. In Section 2, we introduce the theoretical framework underlying our sensitivity analysis method. In Sections 3 and 4, we discuss estimation and interpretation, respectively. Section 5 contains simulation results based on data generated according to three scenarios, and Section 6 presents an application of our sensitivity analysis on a publicly available data set from a randomized controlled trial investigating the effect of umbilical vein oxytocin administration on the need for manual removal of the placenta (Weeks et al. 2010). We end with a discussion on possible extensions of our method.

## 2. THEORETICAL FRAMEWORK

Let $ X $ and $ Y $ be binary random variables representing the treatment and the outcome, respectively. We assume that $ X\,=\,1 $ represents treatment, which is randomized, and that $ Y\,=\,1 $ is the desirable outcome. Let $ Y(x) $ be the potential outcome if the treatment, $ X $, were set to $ x $. We assume consistency holds, so that $ Y(X)=Y $. As the outcome is binary, the expression for the probability of benefit reduces to $ \pi=p\{Y(0)=0, Y(1)=1\} $. Since we cannot observe both potential outcomes for the same individual, the probability of benefit is not identifiable. Nonetheless, sharp nonparametric bounds have been derived in Tian and Pearl (2000), and are given by:


1 \begin{align*}\max(0, p_{1}-p_{0})\leq\pi\leq\min(p_{1},1-p_{0}),\end{align*}

where the marginal distributions, $ p_{1}=p\{Y(1)=1\} $ and $ p_{0}=p\{Y(0)=1\} $, are identified and estimable as $ p(Y\,=\,1\mid X\,=\,1) $ and $ p(Y\,=\,1\mid X\,=\,0) $, respectively.

If $ Y(0)\perp Y(1) $, then $ \pi=p_{1}(1-p_{0})=p(Y\,=\,1\mid X\,=\,1)p(Y\,=\,0\mid X\,=\,0) $, which is identifiable and can be estimated non-parametrically. However, independence between $ Y(0) $ and $ Y(1) $ would almost always be unrealistic, and since the two potential outcomes cannot be observed simultaneously, more information on the joint distribution $ p\{Y(1),Y(0)\} $ is needed in order to compute the probability of benefit. This information can be specified in the form of a sensitivity analysis parameter.

Write $ p_{ab}=p\{Y(0)=a, Y(1)=b\} $, for $ a, b\in\{0,1\} $. For $ p_{10} > 0 $, $ p_{01} > 0 $, $ p_{11} > 0 $ and $ p_{00} > 0 $, let:


2 \begin{align*}\alpha=\log\frac{p_{11}p_{00}}{p_{10}p_{01}}.\end{align*}

Note that any $ (p_{0},p_{1},{p_{ab}}) $, for $ a, b\in\{0,1\} $, determines the joint distribution $ p\{Y(0),Y(1)\} $, since


3 \begin{align*} p_{10}=p_{0}-p_{11},\quad p_{00}=1-p_{1}-p_{0}+p_{11}\quad\mathrm{and}\quad p_{0 1}=p_{1}-p_{11}.\end{align*}

However, none of the $ p_{ab} $ are variation independent of the observed data distribution, since $ p_{ab}\leq\min[p\{Y(0)=a\},p\{Y(1)=b\}] $, so their values are constrained by $ p_{0} $ and $ p_{1} $. Variation independence is an important property of sensitivity analysis parameters, which guarantees that there is no contradiction between a chosen value of the parameter, and the observed data distribution. It also enables effective separation between quantification of uncertainty due to possible deviations from a reference scenario, which the parameter encodes, and quantification of statistical uncertainty. The following result suggests that $ \alpha $ is a convenient sensitivity analysis parameter in this case.

Theorem 1 (Zhang et al. 2013; Rudas 2018). *Suppose $ Y(0) $ and $ Y(1) $ are binary random variables, and suppose $ p_{ab} > 0 $, for all $ a, b\in\{0,1\} $ Then*
 

(1) $ \alpha=0 $  *if and only if* $ Y(0)\perp Y(1) $;
(2) *Given* $ (p_{0},p_{1},\alpha $*), with* $ \alpha\neq 0 $*, we have that:*
 
  4 \begin{align*} p_{11}=\frac{\{(p_{0}+p_{1})(e^{\alpha}-1)+1\}-\sqrt{D}}{2(e^{\alpha}-1)},\end{align*}
  *where*
 
  \begin{align*} D=e^{2\alpha}(p_{0}-p_{1})^{2}+2e^{\alpha}\{p_{0}(1-p_{0})+p_{1}(1-p_{1})\}+(p _{0}+p_{1}-1)^{2} > 0;\end{align*}
(3) *The observed data distribution*, $ p(X, Y) $*, together with*  $ \alpha $*, determine*  $ p\{Y(0),Y(1)\} $;
(4) $ \alpha $  *is variation independent of*  $ p(X, Y) $;
(5) *Among parameters that are variation independent of*  $ p(X, Y) $*, and that together with*  $ p(X, Y) $  *determine*  $ p\{Y(0),Y(1)\} $, $ \alpha $  *is unique, up to a one-to-one transformation.*

The proof of this theorem can be found in standard textbooks, such as Rudas (2018), and is reproduced in Section S1 of the Supplementary Material for completeness. This theorem suggests that $ \alpha $ can be seen as quantifying the deviation from independence between the two potential outcomes $ Y(0) $ and $ Y(1) $, with larger absolute values of $ \alpha $ corresponding to larger deviations. In order to perform sensitivity analysis for the probability of benefit, one might specify a grid for $ \alpha $, and for each value in the grid, recover the probability of benefit as $ \pi=p_{1}-p_{11} $, where $ p_{11} $ is obtained from Equation (4).

To define $ \alpha $, we assume that all $ p_{ab} $, for $ a, b\in\{0,1\} $ are strictly larger than 0. Assuming any of them is exactly equal to 0 is an identifying assumption [cf. relationships in (3)], and makes sensitivity analysis unnecessary. For example, if there is no harm from the treatment, then $ p_{10}=0 $, and so $ \pi=p_{1}-p_{0} $; if there is no benefit from the treatment, then trivially $ \pi\,=\,0 $; if $ p_{11}=0 $, then $ \pi=p_{1} $ and if $ p_{00}=0 $, then $ \pi\,=\,1-p_{0} $. However, there is a potential for problems arising when we do not know that some $ p_{ab}=0 $, and we go ahead and perform sensitivity analysis regardless. In that case, if we let $ \alpha\to\infty $ or $ \alpha\to-\infty $ in the sensitivity analysis, we expect to be able to recover the value of $ \pi $ corresponding to the situation we are in. These limit cases are detailed in Section S2 of the Supplementary Material.

Suppose now that, along with $ X $ and $ Y $, we also measure a vector of baseline covariates, $ Z $, such that


5 \begin{align*} Y(0)\perp Y(1)\mid Z.\end{align*}

Since $ Z $ is measured at baseline, by randomization, we also have that $ \{Y(0),Y(1)\}\perp X\mid Z $, and so


\begin{align*}\pi&=E\Big[p\{Y(1)=1, Y(0)=0\mid Z\}\Big]\\& =E\Big[p\{Y(1)=1\mid Z\}p\{Y(0)=0\mid Z\}\Big]\\& =E\Big[p(Y=1\mid X=1, Z)p(Y=0\mid X=0, Z)\Big],\end{align*}

where $ p(Y\,=\,1\mid X\,=\,1, Z) $ and $ p(Y\,=\,0\mid X\,=\,0, Z) $ can be estimated in a usual way. However, condition (5) cannot be verified in practice and is unlikely to hold in general. The object of our sensitivity analysis is to quantify the deviation from this condition and incorporate this information into an “adjusted” estimate for the probability of benefit.

To perform sensitivity analysis in a general scenario, where we are not guaranteed conditional independence $ Y(0)\perp Y(1)\mid Z $, one might, again, propose a range for $ \alpha $ and proceed as before, ignoring additional information which may come from $ Z $. However, $ \alpha $ quantifies deviation from the stronger condition $ Y(0)\perp Y(1) $, and ignoring information about $ Z $ might lead to wider ranges for $ \alpha $, and consequently, to more conservative intervals for adjusted estimates of the probability of benefit. Moreover, the value of $ \alpha $ that corresponds to conditional independence $ Y(0)\perp Y(1)\mid Z $ would need to be estimated from the observed data, before a grid around it can be specified for the sensitivity analysis. Another straight-forward option would be to perform sensitivity analysis in a stratified manner, by considering conditional odds ratios, which we denote by $ \alpha_{Z} $, defined analogously to (2), but where each occurring probability is conditional on $ Z $. This approach has the advantage that $ \alpha_{Z} $ is variation independent of the observed data distribution $ p(X, Y, Z) $, but may not be feasible for complex, high-dimensional $ Z $, as the number of sensitivity parameters needed would become too large. Instead, we propose to use a marginal sensitivity analysis parameter over $ Z $. Our sensitivity analysis uses the conditional independence $ Y(0)\perp Y(1)\mid Z $ as a reference scenario, under which point identification of the probability of benefit is achieved. The sensitivity analysis is then governed by a parameter that measures deviation from this condition. Specifically, under conditional independence, this parameter has a fixed anchoring point, which does not depend on the observed data, at the value 0, and larger absolute values correspond to larger deviations from conditional independence. To construct this parameter, we contrast a quantity that is unidentified, with its counterpart under the conditional independence assumption $ Y(0)\perp Y(1)\mid Z $.

Let $ x\in\{0,1\} $ be fixed and define


\begin{align*}\delta_{xy}=\alpha_{xy}-\beta_{xy},\end{align*}

where


\begin{align*}\alpha_{xy}=\mathrm{logit}[p\{Y(x)=1\mid Y=y, X=1-x\}],\end{align*}

and


\begin{align*}\beta_{xy}=\mathrm{logit}[E\{p(Y=1\mid X=x, Z)\mid Y=y, X=1-x\}],\end{align*}

for $ y\in\{0,1\} $. It is easy to see that $ \alpha=\alpha_{x1}-\alpha_{x0} $. Moreover, for any $ x\in\{0,1\} $, we have that $ Y(x)\perp Y(1-x)\mid Z $ if and only if $ Y(x)\perp Y\mid(Z, X\,=\,1-x) $ (see Section S3 of the Supplementary Material), so, if $ Y(x)\perp Y(1-x)\mid Z $, then $ \alpha_{xy}=\beta_{xy} $ (see Theorem 2 below). The quantity $ \delta_{xy} $ can thus be described as the difference, on the logit scale, between the probability of the counterfactual outcome $ Y(x) $ being equal to 1 among those who have factually experienced the outcome $ Y\,=\,y $ under $ X\,=\,1-x $, and this same quantity, if we would assume that $ Y(0)\perp Y(1)\mid Z $. The probability of benefit can then be expressed as:


6 \begin{align*} \pi & =p_{01}=p(Y=0\mid X=0)\mathrm{expit}(\delta_{10}+\beta_{10})\end{align*}

7 \begin{align*} & =p(Y=1\mid X=1)\Big\{1-\mathrm{expit}(\delta_{01}+\beta_{01})\Big\}\end{align*}

8 \begin{align*} & =p(Y=1\mid X=1)-p(Y=1\mid X=0)\mathrm{expit}(\delta_{11}+\beta_{11})\end{align*}

9 \begin{align*} & =p(Y=0\mid X=0)-p(Y=0\mid X=1)\Big\{1-\mathrm{expit}(\delta_{00}+\beta_{00})\Big\}.\end{align*}

Note that any two $ \alpha_{xy} $, for $ x, y\in\{0,1\} $ are related via observable quantities, as detailed in the next proposition, whose proof can be found in Section S4 of the Supplementary Material.

Proposition 1 *Let $ X $ and $ Y $ be binary random variables representing the treatment and outcome, respectively, and suppose $ X $ is randomized. Let $ Z $ be a random vector representing measured baseline covariates. Let $ x\in\{0,1\} $ be fixed. For $ y\in\{0,1\} $, write*
 

\begin{align*}\rho_{xy}=\frac{p(Y=1\mid X=x)}{p(Y=y\mid X=1-x)}.\end{align*}

*Then*
 

\begin{align*}\alpha_{x(1-y)}=\mathrm{logit}\Big[\frac{p(Y=y\mid X=1-x)}{p(Y=1-y\mid X=1-x)}\{\rho_{xy}-\mathrm{expit}(\alpha_{xy})\}\Big]\end{align*}

 *and*
 

\begin{align*}\alpha_{(1-x)y}=\mathrm{logit}\Big(\rho_{(1-x)y}[y \ \mathrm{expit}(\alpha_{xy})+(1 -y)\{1-\mathrm{expit}(\alpha_{x(1-y)})\}]\Big).\end{align*}

Since $ \alpha_{xy} $ and $ \delta_{xy} $ are related through the observable quantity $ \beta_{xy} $, it follows that any two distinct $ \delta_{xy} $ are also relatable through observable quantities; in particular,

\begin{align*}\delta_{x(1-y)}=\mathrm{logit}\Big[\frac{p(Y=y\mid X=1-x)}{p(Y=1-y\mid X=1-x)}\{\rho_{xy}-\mathrm{expit}(\delta_{xy}+\beta_{xy})\}\Big]-\beta_{x(1-y)}.\end{align*}

The following theorem, whose proof can be found in Section S5 of the Supplementary Material, establishes the potential of $ \delta_{xy} $ as a sensitivity analysis parameter to quantify deviation from condition (5).

Theorem 2 *Let $ X $ and $ Y $ be binary random variables representing the treatment and outcome, respectively, and suppose $ X $ is randomized. Let $ Z $ be a random vector representing measured baseline covariates. Denote by $ p(X, Y, Z) $ the observed data distribution. Let $ x\in\{0,1\} $ be fixed. Then:*
 

(1) $ \alpha=\alpha_{x1}-\alpha_{x0} $.
(2) *If*  $ Y(x)\perp Z $*, then*  $ \alpha=\delta_{x1}-\delta_{x0}=\delta_{(1-x)1}-\delta_{(1-x)0} $.
(3) *If*  $ Y(1-x)\perp Y(x)\mid Z $*, then*  $ \delta_{xy}=0 $  *for*  $ y\in\{0,1\} $.
(4) *Let*  $ y\in\{0,1\} $*. Given*  $ \delta_{xy} $  *and*  $ p(X, Y, Z) $*, we have that*  $ p_{ab} $  *is identifiable, for any*  $ a, b\in\{0,1\} $.

By Theorem 2  (3), if $ Y(1-x)\perp Y(x)\mid Z $, then $ \delta_{x1}=\delta_{x0}=0 $. While the converse does not generally hold, we expect that in practice, cases where $ \delta_{x1}=\delta_{x0}=0 $ and conditional independence is violated will be rare. To see this, note that if $ \delta_{x1}=\delta_{x0}=0 $, then


\begin{align*} 0&=\int_{Z}[p\{Y(x)=1\mid Z, Y=y, X=1-x\}-p\{Y(x)=1\mid Z, X=x\}]dF_{Z\mid Y=y, X=1-x}\\& =\int_{Z}[p\{Y(x)=1\mid Z, Y=y, X=1-x\}-p\{Y(x)=1\mid Z, X=1-x\}]dF_ {Z\mid Y=y, X=1-x},\end{align*}

for $ y\,=\,0,1 $, where the second equality follows by randomization. However, for this equality to hold in the absence of conditional independence, non-zero probability differences $ p\{Y(x)=1\mid Z, Y\,=\,y, X\,=\,1-x\}-p\{Y(x)=1\mid Z, X\,=\,1-x\} $, would have to cancel out perfectly across levels of $ Z $.

Since $ \mathrm{expit}(\alpha_{10})=p_{01}/(1-p_{0}) $, $ \mathrm{expit}(\alpha_{01})=(p_{1}-p_{01})/p_{1} $, $ \mathrm{expit}(\alpha_{11})=(p_{1}-p_{01})/p_{0} $, $ \mathrm{expit}(\alpha_{00})=(p_{0}-p_{1}+p_{01})/(1-p_{1}) $, the bounds in (1), imply the following bounds for $ \delta_{xy} $:


\begin{align*}\mathrm{logit}\Big\{\frac{\max(0, p_{1}-p_{0})}{1-p_{0}}\Big\}-\beta_{10}&\leq\delta_{10}\leq\mathrm{logit}\Big\{\frac{\min(1-p_{0},p_{1})} {1-p_{0}}\Big\}-\beta_{10}\\\mathrm{logit}\Big\{\frac{p_{1}-\min(1-p_{0},p_{1})}{p_{1}}\Big{\}}-\beta_{01}&\leq\delta_{01}\leq\mathrm{logit}\Big\{\frac{p_{1}-\max(0, p_{1}-p _{0})}{p_{1}}\Big\}-\beta_{01}\\\mathrm{logit}\Big\{\frac{p_{1}-\min(1-p_{0},p_{1})}{p_{0}}\Big{\}}-\beta_{11}&\leq\delta_{11}\leq\mathrm{logit}\Big\{\frac{p_{1}-\max(0, p_{1}-p _{0})}{p_{0}}\Big\}-\beta_{11}\\\mathrm{logit}\Big\{\frac{p_{0}-p_{1}+\max(0, p_{1}-p_{0})}{1-p_{1}}\Big\}-\beta_{00}&\leq\delta_{00}\leq\mathrm{logit}\Big\{\frac{p_{0}-p_{1}+\min(1-p _{0},p_{1})}{1-p_{1}}\Big\}-\beta_{00}.\end{align*}

In particular, if $ p_{1} < 1-p_{0} $, the upper bound for $ \delta_{10} $ is non-trivial, which implies that $ \delta_{10} $ is not variation independent of the observed data distribution. Nonetheless, one of the four $ \delta_{xy} $’s will have unrestricted range, since the bounds become trivial: if $ p_{1}\leq p_{0}\leq 1-p_{1} $, it will be $ \delta_{01} $, if $ 1-p_{0}\leq p_{1}\leq p_{0} $, it will be $ \delta_{10} $, if $ p_{0}\leq p_{1}\leq 1-p_{0} $, it will be $ \delta_{11} $, and if $ 1-p_{1}\leq p_{0}\leq p_{1} $, it will be $ \delta_{00} $. In particular, one of the $ \delta_{xy} $ will be variation independent of $ p(X, Y) $, in the sense that any posited value for that particular $ \delta_{xy} $ is compatible with $ p(X, Y) $. For a formal proof, see Section S6.1 of the Supplementary Material. However, none of the $ \delta_{xy} $ are variation independent of the full observed data distribution $ p(X, Y, Z) $.

Loss of variation independence cannot be circumvented by reverting to the use of $ \alpha $ as a sensitivity analysis parameter, since, in the presence of measured covariates, it is possible to construct examples where $ \alpha $ is not variation independent of the observed data distribution $ p(X, Y, Z) $. In particular, in Section S6.2 of the Supplementary Material, we construct a distribution $ p\{Z, Y(0),Y(1)\} $ that is faithful to the directed acyclic graph $ X\rightarrow Y\leftarrow Z $, and gives rise to an observed data distribution that is incompatible with $ \alpha\,=\,0 $. From this, we argue that there does not exist a parameter that is variation independent of $ p(X, Y, Z) $ and that, together with $ p(X, Y) $, determines $ p\{Y(0),Y(1)\} $. Suppose such a parameter existed, and denote it by $ \vartheta $. Then, since this parameter would also be variation independent of $ p(X, Y) $, by Theorem 1, there would exist a one-to-one map between $ \vartheta $ and $ \alpha $. Let $ \vartheta_{0} $ be the value corresponding to $ \alpha\,=\,0 $ under this map. Then positing $ \vartheta=\vartheta_{0} $ is the same as positing $ \alpha\,=\,0 $, which we are able to reject given the observed data in the above-mentioned example. We state this as a corollary below.

Corollary 1 *Let $ X $ and $ Y $ be binary random variables representing the treatment and outcome, respectively, and suppose $ X $ is randomized. Let $ Y(x) $ be the potential outcome if the treatment, $ X $, were set to $ x $. Let $ Z $ be a random vector representing measured baseline covariates and denote by $ p(X, Y, Z) $ the observed data distribution. Then there does not exist a parameter that is variation independent of $ p(X, Y, Z) $ and that, together with $ p(X, Y) $ determines $ p\{Y(0),Y(1)\} $.*

In practice, this means that for *any* parameter we attempt to use, there is a possibility that certain values for this parameter are not logically compatible with the full observed data distribution $ p(X, Y, Z) $, even if they are logically compatible with $ p(X, Y) $. As a result, the intervals obtained for the probability of benefit may be unnecessarily wide, since they will include values corresponding to values of the sensitivity analysis parameter that should otherwise have been excluded from the grid.

**Fig. 1.**
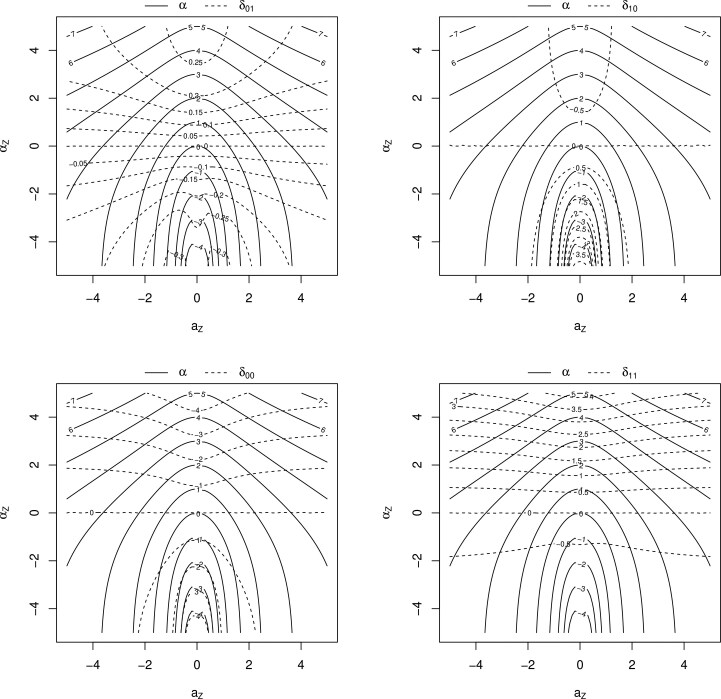
Contours for $ \alpha $ and $ \delta_{xy} $, for $ x, y\in\{0,1\} $, under a joint distribution $ p\{X, Y, Z, Y(0),Y(1)\} $ determined by constant $ \alpha_{Z} $, and observed data distribution $ Z\sim\mathrm{N}(0,1) $, $ X\mid Z\sim\mathrm{Bernoulli}(0.5) $ and $ Y\mid X, Z\sim\mathrm{Bernoulli}\{\mathrm{expit}(2X+a_{Z}Z)\} $. Contours are shown for $ \alpha_{Z} $ and $ a_{Z} $ varying over the interval $ (-5,5) $. For $ \alpha_{Z}=0 $, we have $ Y(0)\perp Y(1)\mid Z $, so $ \delta_{xy}=0 $ for all $ x, y\in\{0,1\} $, but $ \alpha $ may still vary widely.

The advantage of using $ \delta_{xy} $ over $ \alpha $ as a sensitivity analysis parameter is that it incorporates information about $ Z $, and has a natural anchoring point at 0 for the situation where $ Y(0)\perp Y(1)\mid Z $. The corresponding anchoring point for $ \alpha $ is $ \beta_{x1}-\beta_{x0} $, which is a quantity that needs to be estimated from data. The advantage of using $ \delta_{xy} $ over $ \alpha_{Z} $ as a sensitivity analysis parameter is the fact that it is marginal over $ Z $. In particular, it can be used regardless of the dimensionality of $ Z $, without needing to assume that $ \alpha_{Z} $ is constant across levels of $ Z $ or needing to further model $ \alpha_{Z} $ as a function of $ Z $. For further intuition, see Fig. 1, where contour plots of $ \alpha $ and $ \delta_{xy} $ are superimposed, for varying strength of association between $ Y(0) $ and $ Y(1) $ given $ Z $—quantified by the conditional odds ratio $ \alpha_{Z} $, and varying strength of association between $ Y $ and $ Z $, given $ X $. In particular, note that for $ \alpha_{Z}=0 $, all $ \delta_{xy} $ will be equal to zero, but $ \alpha $ may still vary widely.

We have developed the theory for the sensitivity analysis on the logit scale. One reason for doing so was to facilitate the comparison with $ \alpha $. In principle, any link function can be used for the construction of $ \delta_{xy} $. In particular, one might define $ \alpha^{\prime}_{xy}=\mathrm{expit}(\alpha_{xy}) $, $ \beta^{\prime}_{xy}=\mathrm{expit}(\beta_{xy}) $ and $ \delta^{\prime}_{xy}=\alpha^{\prime}_{xy}-\beta^{\prime}_{xy} $. Then if $ Y(1-x)\perp Y(x)\mid Z $, it is still true that $ \delta^{\prime}_{xy}=0 $ for all $ x, y\in\{0,1\} $, and, moreover, if $ p(X, Y, Z) $ and $ \delta^{\prime}_{xy} $ are given, then $ \pi $ is identifiable. However, the range of $ \delta^{\prime}_{xy} $ is restricted to the interval $ (-\beta^{\prime}_{xy},1-\beta^{\prime}_{xy}) $, whose lower and upper limits depend on the observed data.

## 3. ESTIMATION

Let $ n $ be the sample size, consisting of independent and identically distributed observations drawn from $ p(X, Y, Z) $. Let $ x, y\in\{0,1\} $ be fixed, and suppose $ \delta_{xy} $ is given. Then


\begin{align*}\beta_{xy}=\mathrm{logit}E\Bigg\{\mathbb{1}_{Y=y, X=1-x}\frac{p(Y=1\mid X=x, Z)} {p(Y=y, X=1-x)}\Bigg\}\end{align*}

where $ \mathbb{1} $ denotes the indicator function. Hence we can estimate $ \beta_{xy} $ as


10 \begin{align*}\hat{\beta}_{xy}=\mathrm{logit}\Bigg\{\frac{1}{n}\sum\limits_{i=1}^{n}\frac{\mathbb{1}_{Y_{i}=y, X_{i}=1-x}}{\hat{p}(Y=y, X=1-x)}\hat{p}(Y=1\mid X=x, Z_{i})\Bigg\},\end{align*}

where $ \hat{p}(Y\,=\,1\mid X\,=\,x, Z_{i}) $ is a model-based prediction of $ p(Y\,=\,1\mid X\,=\,x, Z_{i}) $ and $ \hat{p}(Y\,=\,y, X\,=\,1-x) $ is a non-parametric estimate of $ p(Y\,=\,y, X\,=\,1-x) $. Together with the given value of $ \delta_{xy} $, the estimate of $ \beta_{xy} $ will allow us to compute the probability of benefit using one of the Equations (6)-(9), where, again, probabilities are replaced by sample counterparts. Because specifying any $ \delta_{xy} $ will suffice to identify the probability of benefit, it may be most convenient to choose the one that is variation independent of $ p(X, Y) $. Alternatively, it may be suitable to choose values of $ x $ and $ y $ for which the number of individuals in the sample with $ Y\,=\,y $ and $ X\,=\,1-x $ is largest, in order to have as many terms as possible contribute to the sum in Equation (10), or values of $ x $ and $ y $ for which subject-matter knowledge can provide a more informed grid for the sensitivity analysis parameter.

If the model for $ p(Y\,=\,1\mid X, Z) $ is correctly specified and the usual regularity conditions hold, then by the theory of M-estimation, $ n^{1/2}(\hat{\pi}-\pi) $ is asymptotically normal with mean 0 and variance given by the sandwich-matrix (Stefanski and Boos 2002). For more details, see Section S7 of the Supplementary Material. It is worth noting that, by specifying a model for $ p(Y\,=\,1\mid X, Z) $, one can obtain $ \beta_{xy} $ for all $ x, y\in\{0,1\} $. Each of these will be consistent and will lead to a consistent estimator of $ \pi $. The resulting estimators of $ \pi $ can then be combined using inverse variance weighting, and the variance of the corresponding combined estimate—which would need to account for the uncertainty in the estimated variances of each individual estimate—can be obtained by using a bootstrap procedure. The relationships in Proposition 1 can be used to transition from one $ \delta_{xy} $ to another, so that the grid for the sensitivity analysis parameter need only be specified for one value of $ x $ and $ y $, even if all parameters are used in the sensitivity analysis. Alternatively, for a chosen $ x $, one might prefer to fit a model $ p(Y\mid X\,=\,x, Z) $ to the subpopulation with $ X\,=\,x $, since it is a model with only $ Z $ as covariate, and does not make any assumptions about $ p(Y\mid X\,=\,1-x, Z) $. Finally, we note that the analyst is not limited to parametric models in the estimation of $ \beta_{xy} $. In principle, any estimation method, including off-the-shelf machine learning methods can be used to model $ p(Y\,=\,1\mid X\,=\,x, Z) $, in which case, confidence intervals may be obtained using bootstrap procedures.

## 4. INTERPRETATION

Proposing a grid for $ \delta_{xy} $ may seem challenging to the analyst, as this parameter is formulated on the logit scale, and furthermore, contrasts two quantities that do not seem easily comparable at first glance. In this case, one may proceed by specifying bounds for a more interpretable quantity, which then translates to bounds for $ \delta_{xy} $ and further, to bounds for the probability of benefit. We will bound the risk difference $ p\{Y(x)=1\mid Z, Y\,=\,y, X\,=\,1-x\}-p\{Y(x)=1\mid Z, Y\,=\,1-y, X\,=\,1-x\} $, but similar results can be obtained using the risk-ratio scale (see Section S8 of the Supplementary Material).

Let


11 \begin{align*} A_{x}=\max\limits_{Z}|p\{Y(x)=1\mid Z, Y=y, X=1-x\}-p\{Y(x)=1\mid Z, Y=1-y, X=1-x\}|.\end{align*}

The quantity $ A_{x} $ contrasts the probability of the counterfactual outcome $ Y(x) $ being equal to 1, among subjects factually exposed to $ X\,=\,1-x $ who have experienced the outcome, versus those factually exposed to $ X\,=\,1-x $ who have not experienced the outcome, across levels of $ Z $. If $ Y(x)\perp Y(1-x)\mid Z $, then $ Y(x)\perp Y\mid(Z, X\,=\,1-x) $ and so $ A_{x}=0 $.

**Fig. 2.**
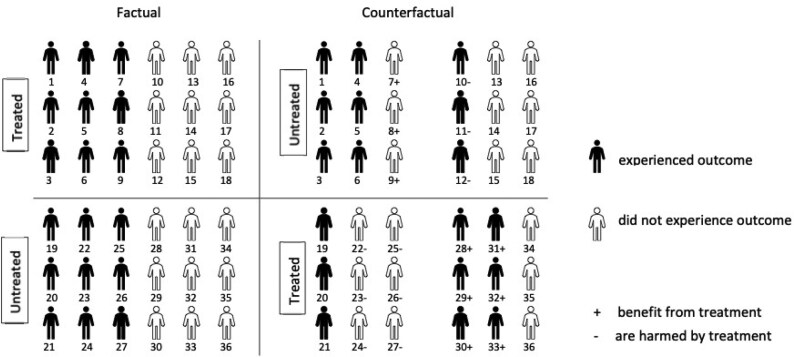
This diagram illustrates 36 subjects, 18 of which are treated and 18 are controls. In this case $ p_{0}=0.5\,=\,p_{1} $, so there is no average causal effect of treatment on the outcome. However, the probability of benefit is equal to 0.25, warranting further investigation into the existence of subpopulations which would profit from receiving the treatment.

For more intuition about $ A_{1} $ and $ A_{0} $, consider the study illustrated in Fig. 2, where there are no baseline covariates. Among patients who were factually treated, patients 1 to 9 experienced the outcome. However, had these patients been untreated, only patients 1 to 6 would have experienced the outcome. Thus, $ p\{Y(0)=1\mid X\,=\,1, Y\,=\,1\}=6/9 $. Similarly, $ p\{Y(0)=1\mid X\,=\,1, Y\,=\,0\}=3/9 $, and so $ A_{0}=0.33 $. Similarly, among patients who were factually untreated, patients 19 to 27 experienced the outcome. However, had these patients been treated, only patients 19 to 21 would have experienced the outcome. Thus, $ p\{Y(1)=1\mid X\,=\,0, Y\,=\,1\}=3/9 $. Similarly, $ p\{Y(1)=1\mid X\,=\,0, Y\,=\,0\}=6/9 $, and so $ A_{1}=0.33 $.

If we define $ \Delta_{xy}=\mathrm{expit}(\alpha_{xy})-\mathrm{expit}(\beta_{xy}) $, then $ -A_{x}\leq\Delta_{xy}\leq A_{x} $ (see Section S8 of the Supplementary Material). Hence, if for a proposed value $ \tilde{A}_{x} $, we have $ \tilde{A}_{x}+\mathrm{expit}(\beta_{xy}) < 1 $, then


12 \begin{align*}\delta_{xy}\leq\mathrm{logit}\{\tilde{A}_{x}+\mathrm{expit}(\beta_{xy})\}-\beta_{xy}.\end{align*}

Similarly, if $ \mathrm{expit}(\beta_{xy})-\tilde{A}_{x} > 0 $, then


13 \begin{align*}\delta_{xy}\geq\mathrm{logit}\{\mathrm{expit}(\beta_{xy})-\tilde{A}_{x}\}-\beta_{xy}.\end{align*}

If one of these conditions does not hold, and an upper or lower limit for the $ \delta_{xy} $-grid cannot be obtained in this way, one may wish to specify an arbitrarily large (respectively, small) value for the failed limit. Alternatively, for more flexibility, one may prefer to specify distinct lower and upper bounds for the probability difference in (11). Details of the resulting bounds for $ \delta_{xy} $ are provided in Section S8 of the Supplementary Material.

It is important to note that there are drawbacks to the use of $ A_{x} $ as a translation quantity for $ \delta_{xy} $. Firstly, the reliability of the transition from the $ A_{x} $-scale to the $ \delta_{xy} $-scale hinges on the correct specification of the model used to estimate $ \beta_{xy} $. Any error introduced in the estimation of $ \beta_{xy} $ will lead to a deviated grid for $ \delta_{xy} $. Moreover, calibrating the grid for $ \delta_{xy} $ using an estimate of $ \beta_{xy} $ from the data at hand results in the blurring between quantification of the deviation from the conditional independence in the reference scenario (5) and quantification of statistical uncertainty. Lastly, specifying a value for $ A_{x} $ will result in an interval for the probability of benefit (via an interval for $ \delta_{xy} $), whereas giving a value for $ \delta_{xy} $ will give a single value for the probability of benefit.

## 5. SIMULATION

We generate observed data according to the following model:


\begin{align*} Z&\sim N(0,1)\\X\mid Z&\sim\mathrm{Bernoulli}(0.5)\\Y\mid X, Z&\sim\mathrm{Bernoulli}\{\mathrm{expit}(a_{0}+a_{X}X+a_{Z}Z)\},\end{align*}

where $ a_{0},a_{X},a_{Z}\in\mathbb{R} $. Note that $ X $ is independent of $ Z $. We next define the conditional odds ratio between $ Y(1) $ and $ Y(0) $ given $ Z $ as a function $ \alpha_{Z}:\mathbb{R}\to\mathbb{R} $. Since $ \alpha_{Z} $ is variation independent of $ p(X, Y\mid Z) $, we can recover the joint distribution $ p\{Y(1),Y(0)\mid Z\} $ using Theorem 1. To compute $ p\{Y(1),Y(0)\} $, we integrate $ p\{Y(1),Y(0)\mid Z\}p(Z) $ over $ Z $.

For our simulation study, we consider three scenarios. In all scenarios, we let $ a_{0}=1 $. In Scenario I, we simulate data so that $ Y(0)\perp Y(1) $. For this, we set $ \alpha_{Z}(z)=0 $, to ensure $ Y(1)\perp Y(0)\mid Z $, and $ a_{Z}=0 $, to ensure $ Y\perp Z\mid X $; we also set $ a_{X}=1 $. In Scenario II, we simulate data under a null average treatment effect, ie $ p\{Y(1)=1\}=p\{Y(0)=1\} $, by setting $ a_{X}=0, a_{Z}=-1 $, and $ \alpha_{Z}(z)=0.2+z $. In Scenario III, we generate data with $ a_{X}=2, a_{Z}=-2 $ and $ \alpha_{Z}(z)=0.2+z $. It is worth noting that, by construction, in Scenario I we have that $ \beta_{x1}=\beta_{x0} $ for $ x\,=\,0,1 $, in Scenario II we have $ \beta_{1y}=\beta_{0y} $ for $ y\,=\,0,1 $, and in Scenario III we have $ \beta_{1y}-\beta_{0y}=a_{X} $ for $ y\,=\,0,1 $. For more details as to why this is the case, see Section S9 of the Supplementary Material.

We generate data for 1,000 populations comprising 500 individuals. In the estimation, we use the correct outcome regression model, ie we fit a logistic regression model with $ X $ and $ Z $ as covariates. To evaluate the performance of our estimation strategy under model misspecification, for the data generated in Scenario III, we fit an outcome regression model with $ X $ and $ \log(|Z|) $ as covariates. We will refer to this as Scenario IIImis.

For each scenario, we present estimation results for all combinations of values for $ x $ and $ y $, using the true value of the associated $ \delta_{xy} $ in the relationships (6)-(9) to obtain an estimate of $ \pi $. We report the mean value, mean estimated standard error and empirical standard error of $ \hat{\pi} $ obtained by using the correct value of $ \delta_{xy} $, for each $ x, y\in\{0,1\} $. We also report the mean value of the associated $ \hat{\beta}_{xy} $ and its empirical standard error. The results of the simulation are contained in Table 1. Plots of the estimated values of the probability of benefit against values of $ \delta_{xy} $ between −5 and 5 can be found in Section S9 of the Supplementary Material.

**Table 1.** Simulation results for 1,000 populations of 500 subjects.^a^

Scenario	$ x, y $	$ \pi $	mean $ \hat{\pi} $	s.e. $ \hat{\pi} $	mean $ \widehat{\mathrm{s.e.}}(\pi) $	cov. prob.	$ \beta_{xy} $	mean $ \hat{\beta}_{xy} $	s.e. $ \hat{\beta}_{xy} $	$ \delta_{xy} $
I	$ x=1, y=0 $	0.237	0.235	0.026	0.025	0.937	2.000	2.014	0.204	0.000
	$ x=0, y=1 $		0.235	0.026	0.025	0.937	1.000	1.015	0.148	0.000
	$ x=1, y=1 $		0.235	0.026	0.025	0.937	2.000	2.027	0.204	0.000
	$ x=0, y=0 $		0.235	0.026	0.025	0.938	1.000	1.002	0.151	0.000
II	$ x=1, y=0 $	0.154	0.153	0.019	0.020	0.943	0.351	0.346	0.172	−0.322
	$ x=0, y=1 $		0.153	0.019	0.019	0.947	1.070	1.084	0.150	0.192
	$ x=1, y=1 $		0.153	0.029	0.029	0.954	1.070	1.070	0.152	0.192
	$ x=0, y=0 $		0.153	0.026	0.027	0.955	0.351	0.356	0.174	−0.322
III	$ x=1, y=0 $	0.246	0.246	0.027	0.027	0.949	1.000	1.009	0.221	−0.157
	$ x=0, y=1 $		0.246	0.026	0.026	0.947	0.867	0.876	0.142	0.063
	$ x=1, y=1 $		0.246	0.034	0.034	0.948	2.867	2.901	0.274	0.409
	$ x=0, y=0 $		0.247	0.033	0.034	0.949	−1.000	−1.013	0.284	−0.503
IIImis	$ x=1, y=0 $	0.246	0.296	0.027	0.027	0.548	1.000	1.845	0.197	−0.157
	$ x=0, y=1 $		0.287	0.028	0.027	0.700	0.867	0.652	0.141	0.063
	$ x=1, y=1 $		0.279	0.030	0.030	0.836	2.867	1.965	0.188	0.409
	$ x=0, y=0 $		0.283	0.029	0.029	0.777	−1.000	0.371	0.170	−0.503

a We report the results of the estimation using all the four possible combinations of values for $ x $ and $ y $, and we use the true value of the corresponding $ \delta_{xy} $. For each scenario and each chosen values of $ x $ and $ y $, we report the true value of $ \pi $, the mean value of $ \hat{\pi} $ (mean $ \hat{\pi} $), the empirical standard error of $ \hat{\pi} $ (s.e. $ \hat{\pi} $), the mean estimated standard error of $ \pi $ (mean $ \widehat{\mathrm{s.e.}}(\pi) $), and the 95% confidence interval coverage probability (cov. prob.) over the 1,000 populations simulated. We also report the true value of $ \beta_{xy} $, the mean value of $ \hat{\beta}_{xy} $ (mean $ \hat{\beta}_{xy} $), the empirical standard error of $ \hat{\beta}_{xy} $ (s.e. $ \hat{\beta}_{xy} $) and the true value of $ \delta_{xy} $. In Scenario IIImis, we use the same data generated for Scenario III, but misspecify the outcome model, by including $ X $ and $ \log(|Z|) $ as covariates, instead of $ X $ and $ Z $.

All estimation results for Scenarios I-III reveal good accuracy, with low bias, low estimated standard errors and confidence interval coverage probabilities close to the nominal value. Both the empirical standard error and the mean estimated standard error for $ \pi $ tended to be larger when $ \delta_{11} $ or $ \delta_{00} $ were used as an input value in the estimation procedure. This can be explained by examining the relationships in (8) and (9), where more quantities need to be estimated than in (6) and (7). When misspecifying the outcome regression model in Scenario IIImis, the estimation results reveal higher bias for $ \pi $ as well as $ \beta_{xy} $, and coverage probabilities below the nominal value for the 95% confidence intervals for $ \hat{\pi} $.

We also evaluated the results of the sensitivity analysis for Scenario III when the quantity $ A_{x} $ was used to arrive at a grid for $ \delta_{xy} $. In particular, for each population, we used the real values of $ A_{0} $ and $ A_{1} $ and the values of $ \hat{\beta}_{xy} $ obtained by fitting the correct model to the data, to obtain grids for $ \delta_{xy} $ using Equations (13) and (12). We note that for $ x\,=\,1 $ the quantities inside the logit function in the expression of the upper bound (12) were larger than 1, so that an arbitrary upper bound for $ \delta_{10} $ and $ \delta_{11} $ was set at 5. Additionally, for $ (x\,=\,0, y\,=\,0) $, in 10% of the populations, the quantities inside the logit function in the expression of the lower bound (13) were negative, so that an arbitrary lower bound for $ \delta_{00} $ was set at $ -5 $ in these cases. Nonetheless, the true value of $ \delta_{xy} $ was covered in 100% of the resulting grids, for all $ x, y\in\{0,1\} $. Further, the true value of $ \pi $ was contained in 99% to 100% of the intervals spanned between the minimum lower 95% CI bound and the maximum upper 95% CI bound over the $ \delta_{xy} $-values in the grid. In contrast, the marginal non-parametric bounds in (1) only cover the true value of $ \pi $ in 74.1% of the cases, and their marginalized counterparts—obtained by estimating the bounds conditional on $ Z $, and then averaging over them (Cai et al. 2007)—cover the true value of $ \pi $ in 75.3% of the cases.

## 6. REAL DATA EXAMPLE

We illustrate our sensitivity analysis by using data from a randomized controlled trial studying umbilical vein oxytocin administration as a treatment for retained placenta (Weeks et al. 2010). The data is publicly available (Weeks et al. 2015). In this study, 577 women who had a retained placenta for more than 30 min were recruited across multiple treatment centers in the UK, Uganda and Pakistan, and randomly assigned to have oxytocin or saline injected into an umbilical vein catheter to the placenta. The outcome of interest was the need for manual removal of the placenta. The study reported a risk ratio of 0.98 (95% CI: 0.87 to 1.12), and therefore concluded that there was no effect of the oxytocin administration on the need for manual removal of the placenta.

Since the more desirable outcome is not having a manual placenta removal, we will code $ Y\,=\,0 $ for a manual placenta removal, and $ Y\,=\,1 $ for other types of placenta delivery. Baseline variables recorded in the study included country in which the treatment center was located, age of the mother, gestational age, previous caeserian section and previous manual removal of placenta, which may be associated with the outcome. We let $ Z $ be the vector of these covariates, which we adjust for in our regression model.

To specify a value for $ A_{1} $, we must speculate about the probability of a manual placenta removal under oxytocin treatment; in particular, we must bound the difference, across all levels of $ Z $, between the counterfactual probability of not having a manual placenta removal procedure among the subjects who factually received the placebo and did not require a manual placenta removal, and those who factually received the placebo and required a manual removal. For the purpose of this analysis, we will posit that this difference is no larger than 0.2 in absolute value, which we consider to be conservative. This amounts to setting $ A_{1}=0.2 $. Similarly, we let $ A_{0}=0.2 $, and its interpretation runs along the same lines: across all levels of $ Z $, if subjects had been administered the placebo, the difference in absolute value between the counterfactual probability of not having a manual placenta removal procedure among the subjects who factually received the oxytocin treatment and did not require a manual placenta removal, and those who factually received the oxytocin treatment and did require a manual removal is no larger than 0.2. This results in the following grids for the values of the sensitivity analysis parameters: $ -1.088\leq\delta_{10}\leq 0.817 $, $ -0.934\leq\delta_{01}\leq 0.813 $, $ -0.974\leq\delta_{11}\leq 0.811 $, $ -1.048\leq\delta_{00}\leq 0.814 $. Since $ \pi $, as a function of $ \delta_{xy} $, is either monotone increasing (for $ y\,=\,0 $) or decreasing (for $ y\,=\,1 $), we only need to examine the endpoints of the grids obtained for $ \delta_{xy} $ in order to obtain the corresponding interval of values for $ \hat{\pi} $. The estimation results can be seen in Table 2. The smallest value of the estimate for $ \pi $ is 0.097 (95% CI: 0.075 to 0.120), and the largest is 0.350 (95% CI: 0.314 to 0.386). This would suggest that between 10% and 35% of the individuals may still benefit from the treatment, which may justify further exploration into the identification of subgroups of patients for which this type of intervention is beneficial.

**Table 2.** Estimation results for the probability of benefit in the real data example, using the extreme values of the grids obtained for $ \delta_{xy} $ by setting $ A_{1}=A_{0}=0.2 $.

$ x, y $	$ \delta_{xy} $	$ \hat{\beta}_{xy} $	$ \hat{\pi} $	95% CI
$ x=1, y=0 $	-1.088	--0.583	0.097	(0.075,0.120)
	0.817		0.343	(0.293,0.393)
$ x=0, y=1 $	0.813	--0.302	0.143	(0.111,0.176)
	-0.934		0.296	(0.249,0.344)
$ x=1, y=1 $	0.811	--0.392	0.150	(0.099,0.202)
	-0.974		0.304	(0.259,0.349)
$ x=0, y=0 $	-1.048	--0.525	0.103	(0.043,0.164)
	0.814		0.350	(0.314,0.386)

The marginal nonparametric bounds for $ \pi $ given in (1), which use no information other than treatment and exposure, result in the interval (0,0.382), which is wider than any of the intervals obtained for $ \hat{\pi} $ using $ \delta_{xy} $, for any $ x, y\in\{0,1\} $. These bounds also constrain $ \delta_{10} $ to the interval $ (-\infty , 1.081) $, $ \delta_{11} $ to $ (-\infty , 5.335) $ and $ \delta_{00} $ to $ (-4.893,1.030) $, but place no restrictions on $ \delta_{01} $. Note that none of the grids posited for $ \delta_{xy} $ lie outside these intervals. The marginalized version of the nonparametric bounds given in (1) result in the interval (0,0.357). Plots of the estimated values of the probability of benefit against values of each $ \delta_{xy} $ for a larger grid covering the interval between −5 and 5 can be found in Section S10 of the Supplementary Material.

The interval obtained for $ \pi $ by using $ \alpha $ as a sensitivity analysis parameter over the grid (−5,5) and ignoring any information on measured covariates is (0.035,0.378). The bounds computed assuming constant $ \alpha_{Z} $ over a grid of values between −5 and 5, as proposed in Zhang et al. (2013), results in an interval for $ \hat{\pi} $ given by (0.038,0.356). This method makes stronger, untestable assumptions about the dependence of the log odds ratio on the measured covariates. In particular, one of the variables in our data is the country of the treatment center, and, in this case, we would be assuming that, the log odds ratio of $ Y(0) $ and $ Y(1) $ is the same for Uganda, Pakistan and the UK, given the same values of the other covariates measured. Nonetheless, a direct comparison between different methods is challenging, because of the different underlying assumptions, and the different considerations for choosing the grid of the sensitivity parameter.

## 7. DISCUSSION

In this paper, we have developed a sensitivity analysis method by quantifying the deviation from conditional independence of the potential outcomes, given a set of measured prognostic baseline covariates. By varying the sensitivity analysis parameter along a grid of values, we obtain a corresponding interval of values for the probability of benefit. Further, for each value in the resulting interval, confidence intervals can be obtained to reflect statistical uncertainty. A drawback of our method is the lack of variation independence between the sensitivity analysis parameters and the full observed data distribution, $ p(X, Y, Z) $. However, in the presence of measured baseline prognostic covariates, we have shown that there does not exist a marginal parameter that, together with the observed data distribution $ p(X, Y) $ identifies the joint distribution $ p\{Y(0),Y(1)\} $ and is variation independent of $ p(X, Y, Z) $. Nonetheless, for any observed data distribution, one of the $ \delta_{xy} $ is variation independent of $ p(X, Y) $. This variation independence can more rigorously be stated as a property of the set of parameters $ \{\delta_{xy}\mid x, y\,=\,0,1\} $ in relation with the observed data distribution $ p(X, Y) $. Specifically, which of the $ \delta_{xy} $ has unconstrained range depends on the data at hand, but any value posited for that $ \delta_{xy} $ will be compatible with the observed distribution $ p(X, Y) $.

In the sensitivity analysis we developed, we favored generality, reflected in the fact that our method does not place any assumption on the functional form or magnitude of the relationships between the exposure, the outcome, and the measured covariates, and the fact that one single parameter can be used regardless of the dimensionality of the measured covariate space. We also strove to provide guidance for interpretation and to describe and evaluate the workflow of our method in practice. However, determining a grid for the sensitivity analysis parameter still requires careful judgment around counterfactual probabilities, informed by subject-matter expertise. Calibrating one’s sensitivity analysis parameters using the data at hand is challenging, as it can blur the separation between quantification of the deviation from the chosen reference scenario, and quantification of statistical uncertainty. Calibration using external data is similarly problematic, and, moreover, will also be plagued by transferability issues.

We have focused on simple randomization as a starting point for our investigation. Variations of the randomization protocol are possible, and our method can be extended to cover these. For example, (possibly stratified) block-randomization, by which we mean randomization to the treatment and control arms so that the proportion of subjects randomized to treatment is the same across blocks, will only affect standard errors, which may be addressed by using a bootstrap approach. For covariate-dependent randomization, where the randomization ratio depends on a subset of variables $ Z^{\prime}\subset Z $, within each level of $ Z^{\prime} $, our method is still valid. In practice, the dimensionality of $ Z^{\prime} $ is expected to be low, so that one can simply perform our proposed sensitivity analysis within strata of $ Z^{\prime} $.

Our sensitivity analysis quantifies deviation from the conditional independence scenario $ Y(0)\perp Y(1)\mid Z $, which allows for the identification of the probability of benefit, and anchors the $ \delta_{xy} $-parameter at 0 in the ensuing analysis. One might be interested in quantifying deviation from another baseline scenario, such as the no-harm scenario, $ Y(1)\geq Y(0) $. In that case, $ p_{10}=0 $, so that $ \alpha_{10}=\mathrm{logit}\{(p_{1}-p_{0})/(1-p_{0})\} $ and $ \alpha_{01}=\mathrm{logit}(p_{0}/p_{1}) $, so for $ x\neq y $, the value of $ \beta_{xy} $ corresponding to this scenario may be estimated from the data, and a grid may be constructed around the resulting value of $ \delta_{xy} $ in the ensuing analysis. However, $ \alpha_{00} $ and $ \alpha_{11} $ are not well defined in this case. This strategy has the disadvantage that it requires the anchoring point of the analysis to be estimated from the data, and renders two of the four possible sensitivity analysis parameters unusable. Alternatively, the no-harm assumption can be incorporated in the bounds for the probability difference involved in the expression for $ A_{x} $.

A natural extension of our method is to consider other types of treatment and outcome variables. For a binary treatment and a categorical outcome with $ K $ levels, one can follow the same approach as outlined here, which will result in $ K-1 $ sensitivity analysis parameters. For categorical $ X $ and $ Y $, with $ K $ and $ L $ levels, respectively, the corresponding representation of the dependence structure is an odds ratio matrix, with each entry corresponding to a given level of $ X $ and a given level of $ Y $. The number of parameters needed in this case would be $ (K-1)(L-1) $. In the continuous outcome case, the natural generalization of the odds ratio is the copula, which allows one to construct a joint cumulative distribution by separately specifying the marginals and the dependence structure between random variables. For non-binary treatment it is not entirely clear how one would define the probability of benefit in a meaningful way.

Another type of extension concerns addressing observational settings, where unmeasured confounders of the treatment and outcome may be present. For this setting, Gabriel et al. (2024) derived sharp non-parametric bounds when the outcome is ordinal. Su and Li (2024) developed a design-based inference method for individual treatment effects in stratified randomized experiments, and further proposed a complementary sensitivity analysis method to evaluate robustness to unmeasured confounding in matched observational studies. Peña (2023) proposed to perform sensitivity analysis on the marginal causal quantities involved in the expression of the sharp non-parametric bounds developed in Tian and Pearl (2000), which incorporate both experimental and observational data. In the same spirit, we believe our previously proposed sensitivitiy analysis method for unmeasured confounding in the estimation of average causal effects (Ciocănea-Teodorescu et al. 2022) can be used together with the method presented in this paper, to provide a sensitivity analysis for the probability of benefit in observational settings susceptible to unmeasured confounding. This analysis would involve two marginal parameters: one that measures deviation from conditional exchangeability of the two potential outcomes given the measured baseline variables, and one that quantifies deviation from conditional exchangeability of the treatment and outcome given measured confounders. Neither of these sensitivity parameters are affected by the dimensionality of measured variables, and no assumptions would be placed on the nature of the unmeasured confounding. This is a subject of further research for the authors.

## Supplementary Material

kxaf011_Supplementary_Data

## Data Availability

The simulation R-code is available at https://github.com/iulianact/senspb.
