# Supplementary material for: Sensitivity analysis for the probability of benefit in randomized controlled trials with a binary treatment and a binary outcome
Source: Biostatistics. 2025 Jun 2;26(1):kxaf011. doi: 10.1093/biostatistics/kxaf011 (PMC12129078; doi:10.1093/biostatistics/kxaf011)
Supplement: kxaf011_Supplementary_Data [file kxaf011_supplementary_data.pdf]

Supplementary material for Sensitivity analysis for the probability of benefit in randomized controlled trials with a binary treatment and a binary outcome

I. CIOCĂNEA-TEODORESCU[1,2], E.E. GABRIEL[3], and A. SJÖLANDER[4]

[1]Victor Babeş National Institute of Pathology, Romania
[2]Carol Davila University of Medicine and Pharmacy, Romania
[3]Section of Biostatistics, Department of Public Health, University of Copenhagen, Denmark
[4]Department of Medical Epidmiology and Biostatistics, Karolinska Institute, Sweden

1 Proof of Theorem 1

Recall that $p_{ab} = p\{Y(0) = a, Y(1) = b\}$, for $a, b \in \{0, 1\}$ and that

$$p_{10} = p_0 - p_{11}, \quad p_{00} = 1 - p_1 - p_0 + p_{11} \quad \text{and} \quad p_{01} = p_1 - p_{11}. \tag{1}$$

1. $\alpha = 0$ if and only if $Y(0) \perp Y(1)$.

   Proof. If $\alpha = 0$, then $p_{11}p_{00} = p_{01}p_{10}$, and the result follows from the relationships displayed in (1). Conversely, if $Y(0) \perp Y(1)$, then $p_{11} = p_0p_1$, $p_{00} = (1 - p_0)(1 - p_1)$, $p_{01} = (1 - p_0)p_1$ and $p_{10} = p_0(1 - p_1)$, so that $\alpha = \log(1) = 0$. □

2. Given (p_0, p_1, α), we have

   $$p_{11} = \frac{\{(p_0 + p_1)(e^\alpha - 1) + 1\} - \sqrt{D}}{2(e^\alpha - 1)},$$

   where $D = e^{2\alpha}(p_0 - p_1)^2 + 2e^\alpha\{p_0(1 - p_0) + p_1(1 - p_1)\} + (p_0 + p_1 - 1)^2$.

   Proof. To see this, let $A = e^\alpha$ and note that, since

   $$A = \frac{p_{11}(1 - p_1 - p_0 + p_{11})}{(p_0 - p_{11})(p_1 - p_{11})}$$

   then

   $$p_{11}^2(A - 1) - p_{11}\{(p_0 + p_1)(A - 1) + 1\} + Ap_0p_1 = 0. \tag{2}$$

   Since $A \neq 1$, the discriminant of quadratic equation (2) is

   $$\begin{aligned} D &= \{(p_0 + p_1)(A - 1) + 1\}^2 - 4A(A - 1)p_0p_1 \\ &= A^2(p_0 - p_1)^2 + 2A\{p_0(1 - p_0) + p_1(1 - p_1)\} + (p_0 + p_1 - 1)^2 \geq 0, \end{aligned}$$

and so the solutions of equation (2) will be of the form

$$p_{11\pm} = \frac{\{(p_0 + p_1)(A-1)+1\} \pm \sqrt{D}}{2(A-1)}.$$

If $A > 1$, since $D \geq A^2(p_0 - p_1)^2$, we have

$$p_{11+} \geq \frac{(p_0 + p_1)(A-1)+1 + A|p_0 - p_1|}{2(A-1)}.$$

Hence, for $p_0 \geq p_1$, we have that $p_{11+} > p_0 \geq p_1$, which lies outside the permitted range of values for p_{11}. By symmetry, for $p_1 \geq p_0$, we have that $p_{11+} > p_1 \geq p_0$.

If $0 < A < 1$, since $D \geq (p_0 + p_1 - 1)^2$, we have

$$p_{11+} \leq \frac{(p_0 + p_1)(1-A) - 1 - |p_0 + p_1 - 1|}{2(1-A)}.$$

For $p_0 + p_1 - 1 \geq 0$, we have

$$p_{11+} \leq \frac{(p_0 + p_1)(1-A) - 1 - (p_0 + p_1 - 1)}{2(1-A)} = -\frac{A(p_0 + p_1)}{2(1-A)} < 0,$$

which is not a permitted value for p_{11}. For $p_0 + p_1 - 1 < 0$, we have

$$p_{11+} \leq \frac{(p_0 + p_1)(1-A) - 1 - (1 - p_0 - p_1)}{2(1-A)} = \frac{2(p_0 + p_1 - 1) - A(p_0 + p_1)}{2(1-A)} < 0,$$

which is, again, not a permitted value for p_{11}. Hence p_{11-} is the only possible solution in the interval $\{0, \min(p_0, p_1)\}$.

Suppose $p_0 > 0$ and $p_1 > 0$, and let $f(x) = x^2(A-1) - x\{(p_0+p_1)(A-1)+1\} + Ap_0p_1$. Then $f(0) = Ap_0p_1 > 0$, $f(p_0) = p_0(p_0 - 1) < 0$ and $f(p_1) = p_1(p_1 - 1) < 0$, so that $f(x)$ must have a solution in the interval $\{0, \min(p_0, p_1)\}$, and by the above argument, it must be p_{11-}. If $p_0 = 0$, then $\sqrt{D} = p_1(A-1) + 1$ and so $p_{11-} = 0$. By symmetry, if $p_1 = 0$, then $\sqrt{D} = p_0(A-1) + 1$, and $p_{11-} = 0$.

□

3. $p(X, Y)$ and α determine $p\{Y(1), Y(0)\}$.

   Proof. Note that the triple (p_0, p_1, p_{11}) determines $p\{Y(1), Y(0)\}$, by the relationships in (1). The result follows from the fact that any given value of α leads to a unique value of p_{11} in the interval $\{0, \min(p_0, p_1)\}$ by the previous result. □

4. α is variation independent of $p(X, Y)$.

   Proof. Let a be any real number. To prove variation independence, we must construct a probability distribution $p^*\{Y(0), Y(1), X, Y\}$ such that

   (a) $Y(X) = Y$ (consistency),

   (b) $\{Y(1), Y(0)\} \perp X$ (randomization),

   (c) $p^*(X, Y) = p(X, Y)$,

(d) $\alpha^* = a$,

where α^* is the log odds ratio of $Y(0)$ and $Y(1)$ under p^*.

We construct $p^*\{Y(0), Y(1), X, Y\}$ by first assuming that $Y(X) = Y$, $\{Y(1), Y(0)\} \perp X$ and $p^*(X, Y) = p(X, Y)$. Then:

$$\begin{aligned} p^*\{Y(X), Y(1-X), X, Y\} &= p^*\{Y(X), Y(1-X), X\} \quad \text{by consistency} \\ &= p^*\{Y(X), Y(1-X)\}p^*(X) \quad \text{by randomization} \\ &= p^*\{Y(X), Y(1-X)\}p(X), \quad \text{since } p^*(X,Y) = p(X,Y). \end{aligned}$$

Note that $p_0^* = p^*\{Y(0)\} = p\{Y(0)\} = p_0$ and $p_1^* = p^*\{Y(1)\} = p\{Y(1)\} = p_1$. Let $A = \exp(a)$. Write $p_{11}^* = p^*\{Y(0) = 1, Y(1) = 1\}$ and note that p_{11}^* is required to take values in the interval $\{0, \min(p_0, p_1)\}$. Set

$$p_{11}^* = \frac{\{(p_0 + p_1)(A-1) + 1\} - \sqrt{D}}{2(A-1)}, \tag{3}$$

with $D = \{(p_0 + p_1)(A-1) + 1\}^2 - 4A(A-1)p_0p_1$, as before. By a previous result, p_{11}^* takes values between 0 and $\min(p_0, p_1)$, and (p_0, p_1, p_{11}^*) determines $p^*\{Y(0), Y(1)\}$ by setting $p_{01}^* = p_1 - p_{11}^*$, $p_{10}^* = p_0 - p_{11}^*$ and $p_{00}^* = 1 - p_1 - p_0 + p_{11}^*$. Moreover, by construction, the log odds ratio between $Y(1)$ and $Y(0)$, $\alpha^* = \log \frac{p_{11}^*(1-p_0-p_1+p_{11}^*)}{(p_0-p_{11}^*(p_1-p_{11}^*))}$, is equal to $\log(A) = a$ by (3). □

5. α is unique, up to a one-to-one transformation.

Proof. Suppose θ is another parameter that is variation independent of $p(X, Y)$ and that together with $p(X, Y)$ determines $p\{Y(0), Y(1)\}$. Since $p(X, Y)$ and α also determine $p\{Y(0), Y(1)\}$, we can express θ as a function of α and $p(X, Y)$, but not of $p(X, Y)$ alone (otherwise θ would not be variation independent). Write this as $\theta = g_{p(X,Y)}(\alpha)$, where the dependence on $p(X, Y)$ is specified in the subscript. Similarly, we can express α as a function of θ and $p(X, Y)$, but not of $p(X, Y)$ alone (otherwise α would not be variation independent). Write this as $\alpha = h_{p(X,Y)}(\theta)$. Then $\theta = g(\alpha) = h(g(\theta))$ and $\alpha = h(\theta) = h(g(\alpha))$ (where we have suppressed the dependence on $p(X, Y)$ in the subscripts) and the conclusion follows.

□

2 Limit cases

Suppose that $p_{10} = 0$ or $p_{01} = 0$, so that α is undefined, but we are unaware of this fact and we go ahead and perform sensitivity analysis. Note that

$$\lim_{\alpha \to \infty} \frac{\{(p_0 + p_1)(e^\alpha - 1) + 1\} - \sqrt{D}}{2(e^\alpha - 1)} = \frac{p_0 + p_1}{2} - \frac{|p_0 - p_1|}{2}.$$

If $p_0 \geq p_1$, then $p_{11} \to p_1$, and conversely, if $p_1 \geq p_0$, then $p_{11} \to p_0$. By the relationships in (1), the former implies $p_{01} \to 0$ and the latter implies that $p_{10} \to 0$. In other words, choosing a large α in the sensitivity analysis corresponds to a no harm or no benefit situation.

Similarly,

$$\lim_{\alpha \to -\infty} \frac{\{(p_0 + p_1)(e^\alpha - 1) + 1\} - \sqrt{D}}{2(e^\alpha - 1)} = \frac{p_0 + p_1 - 1}{2} + \frac{|p_0 + p_1 - 1|}{2}.$$

If $p_0 + p_1 - 1 \geq 0$, then $p_{11} \to p_0 + p_1 - 1$ and by the relationships in (1), $p_{00} \to 0$. If $p_0 + p_1 - 1 \leq 0$, then $p_{11} \to 0$.

3 Auxiliary result

Given $x \in \{0, 1\}$, we have that $Y(x) \perp Y(1-x) \mid Z$ if and only if $Y(x) \perp Y \mid (Z, X = 1-x)$.

Proof. Suppose that $Y(x) \perp Y(1-x) \mid Z$. Then

$$\begin{aligned}
&p\{Y(x) = a, Y = b \mid Z, X = 1-x\} \\
&\quad = p\{Y(x) = a, Y(1-x) = b \mid Z, X = 1-x\} \quad \text{by consistency} \\
&\quad = p\{Y(x) = a, Y(1-x) = b \mid Z\} \quad \text{by randomization} \\
&\quad = p\{Y(x) = a \mid Z\} p\{Y(1-x) = b \mid Z\} \quad \text{by assumption} \\
&\quad = p\{Y(x) = a \mid Z, X = 1-x\} p\{Y(1-x) = b \mid Z, X = 1-x\} \quad \text{by randomization} \\
&\quad = p\{Y(x) = a \mid Z, X = 1-x\} p\{Y = b \mid Z, X = 1-x\} \quad \text{by consistency,}
\end{aligned}$$

and so $Y(x) \perp Y \mid (Z, X = 1-x)$.

Conversely, suppose that $Y(x) \perp Y \mid (Z, X = 1-x)$. Then

$$\begin{aligned}
&p\{Y(x) = a, Y(1-x) = b \mid Z\} \\
&\quad = p\{Y(x) = a, Y(1-x) = b \mid Z, X = 1-x\} \quad \text{by randomization} \\
&\quad = p\{Y(x) = a, Y = b \mid Z, X = 1-x\} \quad \text{by consistency} \\
&\quad = p\{Y(x) = a \mid Z, X = 1-x\} p\{Y = b \mid Z, X = 1-x\} \quad \text{by assumption} \\
&\quad = p\{Y(x) = a \mid Z, X = 1-x\} p\{Y(1-x) = b \mid Z, X = 1-x\} \quad \text{by consistency} \\
&\quad = p\{Y(x) = a \mid Z\} p\{Y(1-x) = b \mid Z\} \quad \text{by randomization.}
\end{aligned}$$

□

4 Proof of Proposition 1

Proof. Note that

$$\begin{aligned}
\operatorname{expit} \alpha_{x(1-y)} &= p\{Y(x) = 1 \mid Y = 1-y, X = 1-x\} \\
&= \frac{p(Y = 1 \mid X = x)}{p(Y = 1-y \mid X = 1-x)} [1 - p\{Y = y \mid Y(x) = 1, X = 1-x\}] \\
&= \frac{p(Y = 1 \mid X = x)}{p(Y = 1-y \mid X = 1-x)} \left[1 - \frac{p(Y = y \mid X = 1-x)}{p(Y = 1 \mid X = x)} p\{Y(x) = 1 \mid Y = y, X = 1-x\}\right] \\
&= \frac{p(Y = y \mid X = 1-x)}{p(Y = 1-y \mid X = 1-x)} \left[\rho_{x,y} - \operatorname{expit} \alpha_{xy}\right].
\end{aligned}$$

Further, since X is randomized, we have that

$$\begin{aligned}
p\{Y(x) = a, Y(1-x) = b\} &= p\{Y(x) = a, Y(1-x) = b \mid X = x\} \\
&= p\{Y = a, Y(1-x) = b \mid X = x\} \\
&= p\{Y(1-x) = b \mid X = x, Y = a\} p(Y = a \mid X = x)
\end{aligned}$$

and, similarly,

$$\begin{aligned}
p\{Y(x) = a, Y(1-x) = b\} &= p\{Y(x) = a, Y(1-x) = b \mid X = 1-x\} \\
&= p\{Y(x) = a, Y = b \mid X = 1-x\} \\
&= p\{Y(x) = a \mid X = 1-x, Y = b\} p(Y = b \mid X = 1-x)
\end{aligned}$$

Hence

$$\frac{p\{Y(1-x)=b \mid X=x, Y=a\}}{p\{Y(x)=a \mid X=1-x, Y=b\}} = \frac{p(Y=b \mid X=1-x)}{p(Y=a \mid X=x)}.$$

In particular,

$$\begin{aligned}
\operatorname{expit}\alpha_{(1-x)y} &= p\{Y(1-x)=1 \mid X=x, Y=y\} \\
&= \frac{p(Y=1 \mid X=1-x)}{p(Y=y \mid X=x)} p\{Y(x)=y \mid X=1-x, Y=1\} \\
&= \rho_{(1-x)y}\{y \operatorname{expit}\alpha_{xy} + (1-y)(1-\operatorname{expit}\alpha_{x(1-y)})\}.
\end{aligned}$$

□

5 Proof of Theorem 2

Proof. 1. By randomization, we may condition on $X = 1-x$ in each of the probabilities appearing in the expression of α, and hence

$$\begin{aligned}
\alpha &= \log \frac{p\{Y(1-x)=1, Y(x)=1 \mid X=1-x\}}{p\{Y(1-x)=0, Y(x)=1 \mid X=1-x\}} \frac{p\{Y(1-x)=0, Y(x)=0 \mid X=1-x\}}{p\{Y(1-x)=1, Y(x)=0 \mid X=1-x\}} \\
&= \log \frac{p\{Y(x)=1 \mid Y=1, X=1-x\}}{p\{Y(x)=1 \mid Y=0, X=1-x\}} \frac{p\{Y(x)=0 \mid Y=0, X=1-x\}}{p\{Y(x)=0 \mid Y=1, X=1-x\}} \\
&= \operatorname{logit}[p\{Y(x)=1 \mid Y=1, X=1-x\}] - \operatorname{logit}[p\{Y(x)=1 \mid Y=0, X=1-x\}] \\
&= \alpha_{x1} - \alpha_{x0},
\end{aligned}$$

where the second equality follows by consistency.

2. Note that if $Y(x) \perp Z$, then $Y \perp Z \mid X = x$, so $\beta_{x1} = \beta_{x0} = \operatorname{logit}\{p(Y=1 \mid X=x)\}$ and then $\delta_{x1} - \delta_{x0} = \alpha_{x1} - \alpha_{x0} = \alpha$. Similarly, $\beta_{(1-x)1} = \beta_{(1-x)0} = \operatorname{logit}\{p(Y(1-x)=1 \mid X=x)\}$ and so $\delta_{(1-x)1} - \delta_{(1-x)0} = \alpha_{(1-x)1} - \alpha_{(1-x)0} = \alpha$.

3. If $Y(1-x) \perp Y(x) \mid Z$, then $Y(x) \perp Y \mid (Z, X=1-x)$, and so, for $y \in \{0,1\}$, we have

$$\begin{aligned}
\alpha_{xy} &= \operatorname{logit}[p\{Y(x)=1 \mid Y=y, X=1-x\}] \\
&= \operatorname{logit} E[p\{Y(x)=1 \mid Y=y, X=1-x, Z\} \mid Y=y, X=1-x] \\
&= \operatorname{logit} E[p\{Y(x)=1 \mid X=1-x, Z\} \mid Y=y, X=1-x] \\
&= \operatorname{logit} E[p\{Y=1 \mid X=x, Z\} \mid Y=y, X=1-x] \\
&= \beta_{xy},
\end{aligned}$$

where the third equality follows by assumption and the fourth equality follows by randomization and consistency.

4. Recall that

$$\begin{aligned}
p_{ab} &= p\{Y(0)=a, Y(1)=b\} \\
&= p\{Y(0)=a, Y(1)=b \mid X=0\} \\
&= p\{Y=a, Y(1)=b \mid X=0\} \\
&= p\{Y(1)=b \mid Y=a, X=0\} p(Y=a \mid X=0) \\
&= p(Y=a \mid X=0)[b \operatorname{expit}\alpha_{1a} + (1-b)(1-\operatorname{expit}\alpha_{1a})].
\end{aligned}$$

Given δ_{xy}, we can compute $\alpha_{xy} = \delta_{xy} + \beta_{xy}$, since β_{xy} is identifiable, and then, if needed, make use of the relationships in Proposition 1 to obtain α_{1a}, which further produces p_{ab}.

□

6 Variation independence

6.1 Existence of a δ_{xy} that is variation independent of $p(X, Y)$

Let X and Y be binary random variables representing treatment and outcome, respectively, and suppose X is randomized. Let $Y(1)$ and $Y(0)$ be the potential outcomes under treatment and control, respectively, with $p_1 = p\{Y(1) = 1\}$ and $p_0 = p\{Y(0) = 1\}$. Further, let Z be a set of measured baseline covariates. Then there exists $x \in \{0, 1\}$ and $y \in \{0, 1\}$ such that δ_{xy} is variation independent of $p(X, Y)$. In particular,

1. if $\max(0, p_1 - p_0) = 0$ and $\min(p_1, 1 - p_0) = p_1$, then δ_{01} is variation independent of $p(X, Y)$;
2. if $\max(0, p_1 - p_0) = 0$ and $\min(p_1, 1 - p_0) = 1 - p_0$, then δ_{10} is variation independent of $p(X, Y)$;
3. if $\max(0, p_1 - p_0) = p_1 - p_0$ and $\min(p_1, 1 - p_0) = p_1$, then δ_{11} is variation independent of $p(X, Y)$;
4. if $\max(0, p_1 - p_0) = p_1 - p_0$ and $\min(p_1, 1 - p_0) = 1 - p_0$, then δ_{00} is variation independent of $p(X, Y)$.

We will provide the proof for the first case. The other cases can be proved along similar lines.

Suppose $\max(0, p_1 - p_0) = 0$ and $\min(p_1, 1 - p_0) = p_1$. Then $p_1 \leq p_0 \leq 1 - p_1$. Let d_{01} be any real number. To prove variation independence, we must construct a probability distribution $p^*\{Y(0), Y(1), X, Y\}$ such that

1. $Y(X) = Y$ (consistency),
2. $(Y(1), Y(0)) \perp X$ (randomization),
3. $p^*(X, Y) = p(X, Y)$,
4. $\delta^*_{01} = d_{01}$,

where

$$\delta^*_{01} = \text{logit}[p^*\{Y(0) = 1 \mid Y = 1, X = 1\}] - \beta^*_{xy},$$

and $\beta^*_{xy} = \text{logit}\, E\{p^*(Y = 1 \mid X = x, Z) \mid Y = y, X = 1 - x\}$

We construct $p^*\{Y(0), Y(1), X, Y\}$ by first assuming that $Y(X) = Y$, $\{Y(1), Y(0)\} \perp X$ and $p^*(X, Y) = p(X, Y)$. Then:

$$\begin{aligned} p^*\{Y(X), Y(1-X), X, Y\} &= p^*\{Y(X), Y(1-X), X\} \quad \text{by consistency} \\ &= p^*\{Y(X), Y(1-X)\}p^*(X) \quad \text{by randomization} \\ &= p^*\{Y(X), Y(1-X)\}p(X) \quad \text{since } p^*(X) = p(X). \end{aligned}$$

Since $p^*(X, Y) = p(X, Y)$ and X is randomized, we also have that $p^*_1 = p_1$, $p^*_0 = p_0$ and $\beta^*_{xy} = \beta_{xy}$, for $x, y \in \{0, 1\}$. Set $p^*_{11} := \text{expit}(d_{01} + \beta_{01})p_1$. Then $0 \leq p^*_{11} \leq p^*_1 = p_1 \leq p_0 = p^*$, by assumption, so it is a well defined probability with values between 0 and $\min(p^*_0, p^*_1)$, which completely determines $p^*\{Y(1), Y(0)\}$ by setting:

$$\begin{aligned} p^*_{01} &= p_1\{1 - \text{expit}(d_{01} + \beta_{01})\}, \\ p^*_{10} &= p_0 - p_1 \text{expit}(d_{01} + \beta_{01}), \\ p^*_{00} &= 1 - p_0 - p_1\{1 - \text{expit}(d_{01} + \beta_{01})\}. \end{aligned}$$

The series of inequalities $p_1 \leq p_0 \leq 1 - p_1$ ensures that all these are well defined. Moreover

$$\begin{aligned} \delta^*_{01} &= \text{logit}[p^*\{Y(0) = 1 \mid Y = 1, X = 1\}] - \beta^*_{01} \\ &= \text{logit}\,\frac{p^*_{11}}{p^*_1} - \beta^*_{01} \\ &= \text{logit}[\text{expit}(d_{01} + \beta_{01})] - \beta_{01} \\ &= d_{01}. \end{aligned}$$

6.2 Loss of variation independence of α in the presence of measured confounders

Let X and Y be binary random variables representing treatment and outcome, respectively, and suppose X is randomized. Let $Y(1)$ and $Y(0)$ be the potential outcomes under treatment and control, respectively. Further, let Z be a binary baseline covariate. Let $p(X, Y, Z)$ be the observed data distribution, and let $\alpha = \log \frac{p_{11}p_{00}}{p_{01}p_{10}}$ be the log odds ratio of the potential outcomes $Y(0)$ and $Y(1)$. We will show that there exists a joint probability distribution $p\{Y(0), Y(1), Z\}$ giving rise to observed data, for which the value $\alpha = 0$ can be rejected. Thus, α is not variation independent of the observed data distribution.

Suppose α is given, and let $p\{X, Y, Z\}$ be the observed distribution. Then $p\{Y(0), Z\} = p(Y, Z \mid X = 0)$, $p\{Y(1), Z\} = p(Y, Z \mid X = 1)$, $p\{Y(0)\} = p(Y \mid X = 0)$ and $p\{Y(1)\} = p(Y \mid X = 1)$ are all observed. Hence, the values of $p\{Z \mid Y(0)\}$, $p\{Z \mid Y(1)\}$ are also determined. By Theorem 1 of the main text, the triple $\{\alpha, p\{Y(0)\}, p\{Y(1)\}\}$ determines $p\{Y(0), Y(1)\}$. Note that, by the law of total probability,

$$p\{Z \mid Y(0)\} = \sum_{y_1 \in \{0,1\}} p\{Z \mid Y(0), Y(1) = y_1\} p\{Y(1) = y_1 \mid Y(0)\} \tag{4}$$

and

$$p\{Z \mid Y(1)\} = \sum_{y_0 \in \{0,1\}} p\{Z \mid Y(0) = y_0, Y(1)\} p\{Y(0) = y_0 \mid Y(1)\}. \tag{5}$$

In these expressions, all quantities except $p\{Z \mid Y(0) = y_0, Y(1)\}$ and $p\{Z \mid Y(0), Y(1) = y_1\}$ are determined by α and the observed data distribution. We will show that there do not always exist values of $p\{Z \mid Y(0) = y_0, Y(1)\}$ and $p\{Z \mid Y(0), Y(1) = y_1\}$ that satisfy (4) and (5).

For this, define

$$\begin{aligned}
u_{y_0} &= p\{Z = 1 \mid Y(0) = y_0\} \\
v_{y_1} &= p\{Z = 1 \mid Y(1) = y_1\} \\
w_{y_0 y_1} &= p\{Z = 1 \mid Y(0) = y_0, Y(1) = y_1\} \\
p_{y_0 y_1} &= p\{Y(0) = y_0, Y(1) = y_1\} \\
p_{\cdot y_1} &= p_{0 y_1} + p_{1 y_1} \\
p_{y_0 \cdot} &= p_{y_0 0} + p_{y_0 1}.
\end{aligned}$$

Then, by (4) and (5), we have that

$$\begin{aligned}
u_0 &= \frac{w_{00}p_{00} + w_{01}p_{01}}{p_{0\cdot}} \\
v_0 &= \frac{w_{00}p_{00} + w_{10}p_{10}}{p_{\cdot 0}}
\end{aligned}$$

which further implies that

$$w_{10} = \frac{v_0 p_{\cdot 0} - u_0 p_{0\cdot} + w_{01}p_{01}}{p_{10}} \geq \frac{v_0 p_{\cdot 0} - u_0 p_{0\cdot}}{p_{10}}. \tag{6}$$

The right-hand-side of the inequality in (6), need not be less than or equal to 1.

Consider the counterfactual distribution defined by the following probabilities:

$$p\{Z=0,Y(0)=0,Y(1)=0\}=0.15,$$
$$p\{Z=0,Y(0)=0,Y(1)=1\}=0.23,$$
$$p\{Z=0,Y(0)=1,Y(1)=0\}=0.01,$$
$$p\{Z=0,Y(0)=1,Y(1)=1\}=0.01,$$
$$p\{Z=1,Y(0)=0,Y(1)=0\}=0.2,$$
$$p\{Z=1,Y(0)=0,Y(1)=1\}=0.03,$$
$$p\{Z=1,Y(0)=1,Y(1)=0\}=0.3,$$
$$p\{Z=1,Y(0)=1,Y(1)=1\}=0.07.$$

Then the observed quantities are $u_0 = p\{Z = 1 \mid Y(0) = 0\} = 0.377$, $u_1 = p\{Z = 1 \mid Y(0) = 1\} = 0.949$, $v_0 = p\{Z = 1 \mid Y(1) = 0\} = 0.758$, $v_1 = p\{Z = 1|Y(1) = 1\} = 0.294$. It can also be verified that the true value of α is -1.057. If we were to posit $\alpha = 0$, then the implied value of p_{10} would be $p_{1\cdot}p_{\cdot 0} = 0.257$, and so the lower bound in (6) would be equal to 1.049, which is a contradiction. In particular, we have been able to reject marginal independence of the potential outcomes $Y(0)$ and $Y(1)$.

7 Estimation

Write $q_{xy} = p(X = x, Y = y)$, $r_x = p(X = x)$ and let $m(Z, X; \gamma)$ be the model specified for $p(Y = 1 \mid X, Z)$. Let $S_i(\gamma)$ be an estimating function for γ under this model, with $E\{S_i(\gamma)\} = 0$, and write $m_x(Z; \gamma) = m(Z, X = x; \gamma)$.

Estimates of π using a given value for δ_{xy}, with $x \neq y$, can be obtained by solving the set of estimating equations $\sum_{i=1}^n U_i^{xy}(\theta^{xy})$=0, where

$$U_i^{xy}(\theta^{xy}) = \begin{bmatrix} \mathbb{1}_{X_i=1-x, Y_i=y} - q_{(1-x)y} \\ \mathbb{1}_{X_i=1-x} - r_{1-x} \\ S_i(\gamma) \\ \frac{\mathbb{1}_{X_i=1-x,Y_i=y}}{q_{(1-x)y}} m_x(Z_i;\gamma) - \operatorname{expit}\beta_{xy} \\ \frac{q_{(1-x)y}}{r_{1-x}}[x \operatorname{expit}(\beta_{xy}+\delta_{xy}) + (1-x)\{1-\operatorname{expit}(\beta_{xy}+\delta_{xy}\})] - \pi \end{bmatrix},$$

where $\theta^{xy} = (q_{(1-x)y}, r_{1-x}, \gamma, \beta_{xy}, \pi)$ and $\mathbb{1}$ is the indicator function.

Estimates of π using a given value for δ_{xy}, with $x = y$, can be obtained by solving the set of estimating equations $\sum_{i=1}^n U_i^{xy}(\theta^{xy})$=0, where

$$U_i^{xy}(\theta^{xy}) = \begin{bmatrix} \mathbb{1}_{X_i=1-x, Y_i=y} - q_{(1-x)y} \\ \mathbb{1}_{X_i=x, Y_i=y} - q_{xy} \\ \mathbb{1}_{X_i=1-x} - r_{1-x} \\ S_i(\gamma) \\ \frac{\mathbb{1}_{X_i=1-x,Y_i=y}}{q_{(1-x)y}} m_x(Z_i;\gamma) - \operatorname{expit}\beta_{xy} \\ \frac{q_{xy}}{1-r_{1-x}} - \frac{q_{(1-x)y}}{r_{1-x}}[x \operatorname{expit}(\beta_{xy}+\delta_{xy}) + (1-x)\{1-\operatorname{expit}(\beta_{xy}+\delta_{xy})\}] - \pi \end{bmatrix},$$

where $\theta^{xy} = (q_{(1-x)y}, q_{xy}, r_{1-x}, \gamma, \beta_{xy}, \pi)$.

Alternatively, separate models $m_0(Z; \gamma_0)$ and $m_1(Z; \gamma_1)$ can be specified for $p(Y = 1 \mid X = 0, Z)$ and $p(Y = 1 \mid X = 1, Z)$, respectively.

Let $A^{xy}(\theta^{xy}) = E\Big\{-\frac{\partial U_i^{xy}}{\partial \theta^{xy\top}}(\theta^{xy})\Big\}$ and $B^{xy}(\theta^{xy}) = \operatorname{var}\{U_i^{xy}(\theta^{xy})\}$. Then the sandwich variance matrix is given by $A^{xy}(\theta^{xy})^{-1}B^{xy}(\theta^{xy})A^{xy}(\theta^{xy})^{-\top}$.

8 Interpretation

Let

$$\begin{aligned}\Delta_{xy} &= p\{Y(x)=1 \mid Y=y, X=1-x\} - E\{p(Y=1 \mid X=x, Z) \mid Y=y, X=1-x\} \\ &= \operatorname{expit}\alpha_{xy} - \operatorname{expit}\beta_{xy}.\end{aligned}$$

Note that

$$\begin{aligned}&p\{Y(x)=1 \mid X=1-x, Y=y, Z\} - p\{Y(x)=1 \mid X=1-x, Z\} \\ &= p\{Y(x)=1 \mid X=1-x, Y=y, Z\} - p\{Y(x)=1 \mid X=1-x, Y=y, Z\}p(Y=y \mid X=1-x, Z)- \\ &\quad - p\{Y(x)=1 \mid X=1-x, Y=1-y, Z\}p(Y=1-y \mid X=1-x, Z) \\ &= p\{Y(x)=1 \mid X=1-x, Y=y, Z\}[1-p(Y=y \mid X=1-x, Z)]- \\ &\quad - p\{Y(x)=1 \mid X=1-x, Y=1-y, Z\}p(Y=1-y \mid X=1-x, Z) \\ &= p\{Y(x)=1 \mid X=1-x, Y=y, Z\}p(Y=1-y \mid X=1-x, Z)- \\ &\quad - p\{Y(x)=1 \mid X=1-x, Y=1-y, Z\}p(Y=1-y \mid X=1-x, Z) \\ &= [p\{Y(x)=1 \mid X=1-x, Y=y, Z\} - p\{Y(x)=1 \mid X=1-x, Y=1-y, Z\}]\cdot \\ &\quad \cdot p(Y=1-y \mid X=1-x, Z) \qquad (7)\end{aligned}$$

and so

$$\begin{aligned}\Delta_{xy} &= \sum_Z [p\{Y(x)=1 \mid Y=y, X=1-x, Z\} - p\{Y(x)=1 \mid X=x, Z\}]p(Z \mid Y=y, X=1-x) \\ &= \sum_Z [p\{Y(x)=1 \mid Y=y, X=1-x, Z\} - p\{Y(x)=1 \mid X=1-x, Z\}]p(Z \mid Y=y, X=1-x) \\ &= \sum_Z [p\{Y(x)=1 \mid Y=y, X=1-x, Z\} - p\{Y(x)=1 \mid X=1-x, Y=1-y, Z\}]\cdot \\ &\quad \cdot p(Y=1-y \mid X=1-x, Z)p(Z \mid Y=y, X=1-x),\end{aligned}$$

where the second equality follows by randomization, and the third follows by (7).

Let

$$A_x = \max_Z |p\{Y(x)=1 \mid X=1-x, Y=y, Z\} - p\{Y(x)=1 \mid X=1-x, Y=1-y, Z\}|.$$

Then $A_x \geq 0$ and

$$-A_x \leq p\{Y(x)=1 \mid X=1-x, Y=y, Z\} - p\{Y(x)=1 \mid X=1-x, Y=1-y, Z\} \leq A_x,$$

for all Z, so

$$\Delta_{xy} \leq A_x \sum_Z p(Y=1-y \mid X=1-x, Z)p(Z \mid Y=y, X=1-x) \leq A_x.$$

Similarly,

$$\Delta_{xy} \geq -A_x \sum_Z p(Y=1-y \mid X=1-x, Z)p(Z \mid Y=y, X=1-x) \geq -A_x.$$

Hence $-A_x \leq \Delta_{xy} \leq A_x$.

Alternatively, define:

$$A_{xy}^{\max} = \max_Z \left[p\{Y(x) = 1 \mid Z, Y = y, X = 1-x\} - p\{Y(x) = 1 \mid Z, Y = 1-y, X = 1-x\}\right],$$
$$A_{xy}^{\min} = \min_Z \left[p\{Y(x) = 1 \mid Z, Y = y, X = 1-x\} - p\{Y(x) = 1 \mid Z, Y = 1-y, X = 1-x\}\right].$$

Then

$$A_{xy}^{\min} \leq p\{Y(x) = 1 \mid Z, Y = y, X = 1-x\} - p\{Y(x) = 1 \mid Z, Y = 1-y, X = 1-x\} \leq A_{xy}^{\max}$$

for all Z.

If $A_{xy}^{\max} \geq 0$, we have that

$$\Delta_{xy} \leq A_{xy}^{\max} \sum_Z p(Y = 1-y \mid X = 1-x, Z) p(Z \mid Y = y, X = 1-x) \leq A_{xy}^{\max}.$$

Since

$$\begin{aligned}
\Delta_{xy} &\geq A_{xy}^{\min} \sum_Z p(Y = 1-y \mid X = 1-x, Z) p(Z \mid Y = y, X = 1-x) \\
&= A_{xy}^{\min} \sum_Z \{1 - p(Y = y \mid X = 1-x, Z)\} p(Z \mid Y = y, X = 1-x) \\
&= A_{xy}^{\min} \left[1 - \sum_Z p(Y = y \mid X = 1-x, Z) \frac{p(Y = y \mid Z, X = 1-x) p(Z, X = 1-x)}{p(Y = y, X = 1-x)}\right],
\end{aligned}$$

if $A_{xy}^{\min} \leq 0$, then

$$\Delta_{xy} \geq A_{xy}^{\min}.$$

Otherwise, if $A_{xy}^{\min} \geq 0$, then

$$\Delta_{xy} \geq A_{xy}^{\min} \left\{1 - \frac{1}{p(Y = y \mid X = 1-x)}\right\}.$$

Similarly, if $A_{xy}^{\max} < 0$, then $A_{xy}^{\min} < 0$, and we have that

$$\Delta_{xy} \geq A_{xy}^{\min} \sum_Z p(Y = 1-y \mid X = 1-x, Z) p(Z \mid Y = y, X = 1-x) \geq A_{xy}^{\min}$$

and

$$\begin{aligned}
\Delta_{xy} &\leq A_{xy}^{\max} \sum_Z p(Y = 1-y \mid X = 1-x, Z) p(Z \mid Y = y, X = 1-x) \\
&\leq A_{xy}^{\max} \left\{1 - \frac{1}{p(Y = y \mid X = 1-x)}\right\}
\end{aligned}$$

Analogously, for the risk ratio scale, define

$$C_{xy}^{\max} = \max_Z \frac{p\{Y(x) = 1 \mid Z, Y = y, X = 1-x\}}{p\{Y(x) = 1 \mid Z, Y = 1-y, X = 1-x\}},$$
$$C_{xy}^{\min} = \min_Z \frac{p\{Y(x) = 1 \mid Z, Y = y, X = 1-x\}}{p\{Y(x) = 1 \mid Z, Y = 1-y, X = 1-x\}}.$$

Then

$$
\begin{aligned}
(C_{xy}^{\min} - 1)p\{Y(x) = 1 \mid Z, Y = 1-y, X = 1-x\} &\leq \\
&\leq p\{Y(x) = 1 \mid Z, Y = y, X = 1-x\} - p\{Y(x) = 1 \mid Z, Y = 1-y, X = 1-x\} \\
&\leq (C_{xy}^{\max} - 1)p\{Y(x) = 1 \mid Z, Y = 1-y, X = 1-x\}
\end{aligned}
$$

for all Z.

If $C_{xy}^{\max} \geq 1$, we have that

$$
\begin{aligned}
\Delta_{xy} &\leq (C_{xy}^{\max} - 1) \sum_Z \Big[p\{Y(x) = 1 \mid Z, Y = 1-y, X = 1-x\}p(Y = 1-y \mid X = 1-x, Z)\cdot \\
&\qquad \cdot p(Z \mid Y = y, X = 1-x)\Big] \\
&\leq C_{xy}^{\max} - 1.
\end{aligned}
$$

Since

$$
\begin{aligned}
\Delta_{xy} \geq (C_{xy}^{\min} - 1) \sum_Z \Big[p\{Y(x) = 1 \mid Z, Y = 1-y, X = 1-x\}p(Y = 1-y \mid X = 1-x, Z)\cdot \\
\cdot p(Z \mid Y = y, X = 1-x)\Big],
\end{aligned}
$$

if $C_{xy}^{\min} \leq 1$, then

$$
\Delta_{xy} \geq C_{xy}^{\min} - 1.
$$

Otherwise, if $C_{xy}^{\min} \geq 1$, then

$$
\begin{aligned}
\Delta_{xy} &\geq \frac{C_{xy}^{\min} - 1}{p(Y = y, X = 1-x)} \sum_Z \Big[p\{Y(x) = 1 \mid Z, Y = 1-y, X = 1-x\}p(Z, Y = 1-y, X = 1-x)\cdot \\
&\qquad \cdot \{1 - p(Y = 1-y \mid X = 1-x, Z)\}\Big]. \\
&= \frac{C_{xy}^{\min} - 1}{p(Y = y, X = 1-x)} \Big[p\{Y(x) = 1, Y(1-x) = 1-y, X = 1-x\} - \\
&- \sum_Z p\{Y(x) = 1 \mid Z, Y = 1-y, X = 1-x\}p(Y = 1-y \mid X = 1-x, Z)p(Z, Y = 1-y, X = 1-x)\Big]\Big\} \\
&\geq \frac{C_{xy}^{\min} - 1}{p(Y = y, X = 1-x)} \Big[p\{Y(x) = 1, Y(1-x) = 1-y, X = 1-x\} - p(Y = 1-y, X = 1-x)\Big] \\
&\geq (1 - C_{xy}^{\min}) \frac{p(Y = 1-y, X = 1-x)}{p(Y = y, X = 1-x)}.
\end{aligned}
$$

Similarly, if $C_{xy}^{\max} < 1$, then $C_{xy}^{\min} < 1$, and we have that

$$
\Delta_{xy} \leq (1 - C_{xy}^{\max}) \frac{p(Y = 1-y, X = 1-x)}{p(Y = y, X = 1-x)} \tag{8}
$$

and

$$
\Delta_{xy} \geq (C_{xy}^{\min}) - 1. \tag{9}
$$

Once bounds for Δ_{xy} have been established, they translate to bounds for α_{xy}, which, given the observed data, further translate to bounds for δ_{xy}.

9 Simulation

The estimates of π as a function of δ_{xy}, for $x, y \in \{0, 1\}$, for simulations Scenarios I-III, can be seen in Figures 1-3, respectively. For each scenario, estimates are obtained from a population of size 10 000 and the grid used for δ_{xy} is the interval (-5,5).

9.1 Scenario I

In Scenario I, we have $\alpha = 0$ and $\delta_{xy} = 0$, for all $x, y \in \{0, 1\}$, so that $\alpha_{xy} = \beta_{xy}$. Since $\alpha = \alpha_{x1} - \alpha_{x0}$, it follows that $\beta_{11} = \beta_{10}$ and $\beta_{01} = \beta_{00}$.

9.2 Scenario II

In Scenario I, we have $a_X = 0$ and $p_0 = p_1$. Hence

$$\begin{aligned}
\beta_{11} &= \text{logit} \sum_Z p(Y = 1 \mid X = 1, Z) p(Z \mid Y = 1, X = 0) \\
&= \text{logit} \sum_Z p(Y = 1 \mid X = 1, Z) \frac{p(Y = 1 \mid Z, X = 0) p(Z)}{p(Y = 1 \mid X = 0)} \\
&= \text{logit} \sum_Z p(Y = 1 \mid X = 0, Z) \frac{p(Y = 1 \mid Z, X = 1) p(Z)}{p(Y = 1 \mid X = 1)} \\
&= \text{logit} \sum_Z p(Y = 1 \mid X = 0, Z) p(Z \mid Y = 1, X = 1) \\
&= \beta_{01}.
\end{aligned}$$

Similarly, $\beta_{10} = \beta_{00}$.

9.3 Scenario III

Note that

$$\begin{aligned}
\beta_{10} &= \text{logit} \sum_Z p(Y = 1 \mid X = 1, Z) \frac{p(Y = 0 \mid X = 0, Z) p(Z)}{p(Y = 0 \mid X = 0)} \\
&= \text{logit} \left[\frac{1}{p(Y = 0 \mid X = 0)} \sum_Z p(Y = 1 \mid X = 1, Z) \{1 - p(Y = 1 \mid X = 0, Z)\} p(Z) \right] \\
&= \text{logit} \left[\frac{1}{p(Y = 0 \mid X = 0)} \left\{ \sum_Z p(Y = 1 \mid X = 1, Z) p(Z \mid X = 1) - \right.\right. \\
&\qquad \left.\left. - \sum_Z p(Y = 1 \mid X = 1, Z) p(Y = 1 \mid X = 0, Z) p(Z) \right\} \right].
\end{aligned}$$

Let

$$S = \sum_Z p(Y = 1 \mid X = 1, Z) p(Y = 1 \mid X = 0, Z) p(Z).$$

Then

$$\beta_{10} = \text{logit} \left(\frac{p_1 - S}{1 - p_0} \right),$$

and, similarly,

$$\beta_{00} = \operatorname{logit}\left(\frac{p_0 - S}{1 - p_1}\right).$$

Hence

$$\beta_{10} - \beta_{00} = \log\left(\frac{p_1 - S}{1 - p_0 - p_1 + S} \cdot \frac{1 - p_1 - p_0 + S}{p_0 - S}\right) = \log\left(\frac{p_1 - S}{p_0 - S}\right).$$

Further,

$$\begin{aligned}
p_1 - S &= p(Y = 1 \mid X = 1) - \sum_Z p(Y = 1 \mid X = 1, Z)p(Y = 1 \mid X = 0, Z)p(Z) \\
&= \sum_Z p(Y = 1 \mid X = 1, Z)\{1 - p(Y = 1 \mid X = 0, Z)\}p(Z) \\
&= \sum_Z p(Y = 1 \mid X = 1, Z)p(Y = 0 \mid X = 0, Z)p(Z) \\
&= \sum_Z \operatorname{expit}(a_0 + a_X + a_Z Z)\{1 - \operatorname{expit}(a_0 + a_Z Z)\}p(Z) \\
&= \sum_Z \frac{e^{a_0+a_X+a_Z Z}}{1 + e^{a_0+a_X+a_Z Z}}\left(1 - \frac{e^{a_0+a_Z Z}}{1 + e^{a_0+a_Z Z}}\right)p(Z) \\
&= e^{a_X} \sum_Z \frac{e^{a_0+a_Z Z}}{(1 + e^{a_X} e^{a_0+a_Z Z})(1 + e^{a_0+a_Z Z})}p(Z).
\end{aligned}$$

Similarly, it can be shown that

$$p_0 - S = \sum_Z \frac{e^{a_0+a_Z Z}}{(1 + e^{a_X} e^{a_0+a_Z Z})(1 + e^{a_0+a_Z Z})}p(Z),$$

and so $\log\left(\frac{p_1-S}{p_0-S}\right) = \log(e^{a_X}) = a_X$.

10 Real data example

The estimates of π as a function of δ_{xy}, for $x, y \in \{0, 1\}$, for the Release randomized controlled study data, can be seen in Figure 4. The grid used for δ_{xy} is the interval (-5,5). The values of $\hat{\pi}$ obtained when using δ_{01} and δ_{11}, and those obtained using δ_{10} and δ_{00} are seen to be nearly identical.

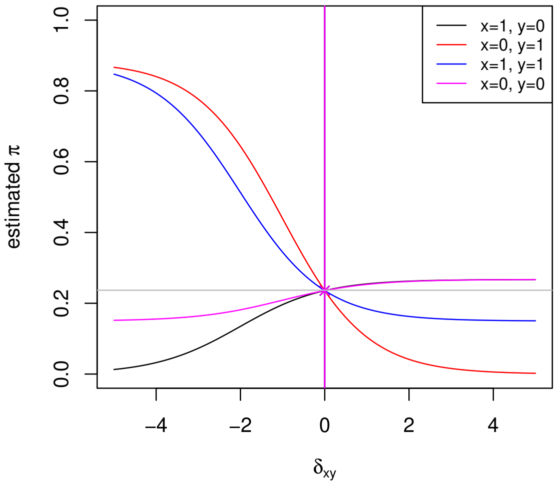

Figure 1: The estimates of π as a function of δ_{xy}, for $x, y \in \{0, 1\}$ for Scenario I. The horizontal grey line corresponds to the true value of π. The vertical lines correspond to the true value of δ_{xy}, which in this case are all equal. The values of $\hat{\pi}$ corresponding to the true value of δ_{xy} are marked with a $\times$-symbol, which in this case overlap.

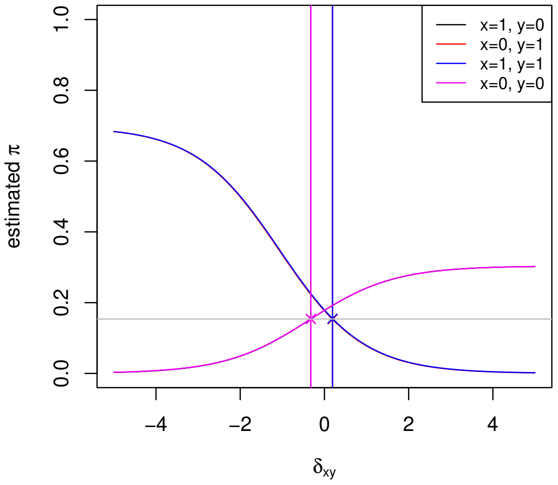

Figure 2: The estimates of π as a function of δ_{xy}, for $x, y \in \{0, 1\}$ for Scenario II. The horizontal grey line corresponds to the true value of π. The vertical lines correspond to the true value of δ_{xy}, which in this case overlap for $x = 1, y = 1$ and $x = 0, y = 1$, and for $x = 1, y = 0$ and $x = 0, y = 0$. The values of $\hat{\pi}$ corresponding to the true value of δ_{xy} are marked with a $\times$-symbol, which in this case overlap for $x = 1, y = 1$ and $x = 0, y = 1$, and for $x = 1, y = 0$ and $x = 0, y = 0$.

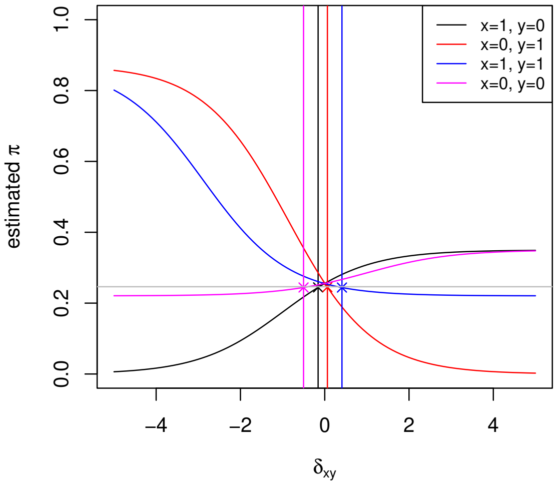

Figure 3: The estimates of π as a function of δ_{xy}, for $x, y \in \{0, 1\}$ for Scenario III. The horizontal grey line corresponds to the true value of π. The vertical lines correspond to the true value of δ_{xy}. The values of $\hat{\pi}$ corresponding to the true value of δ_{xy} are marked with a $\times$-symbol.

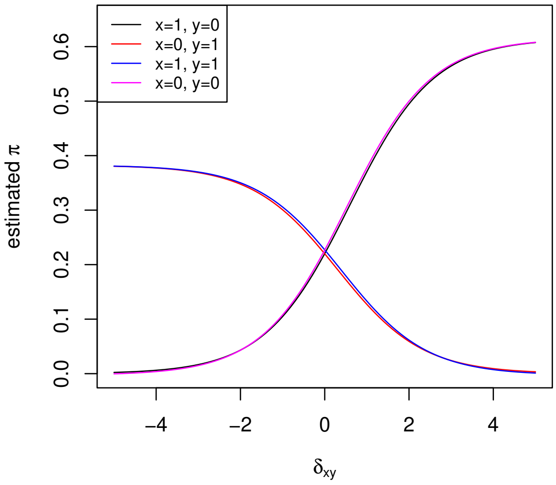

Figure 4: The estimates of π as a function of δ_{xy}, for $x, y \in \{0, 1\}$ for the Release randomized controlled trial data.
